# What Tools Do We Have to End the US HIV Epidemic? A Review of Structural, Biomedical and Implementation Interventions and Their Potential Population Impact

**DOI:** 10.1002/jia2.70146

**Published:** 2026-07-09

**Authors:** Micah Piske, Brenda Carolina Guerra‐Alejos, Minh Tri Van, Benjamin Enns, Melanie G. Medina, Xiao Zang, Wendy S. Armstrong, Czarina Behrends, Carlos del Rio, Eva Enns, Elvin H. Geng, Matthew R. Golden, Brandon D. L. Marshall, Shruti H. Mehta, Lisa R. Metsch, Bruce R. Schackman, Steffanie A. Strathdee, Hansel E. Tookes, Bohdan Nosyk, the Localized Economic Modeling group

**Affiliations:** ^1^ Centre for Advancing Health Outcomes Vancouver British Columbia Canada; ^2^ Faculty of Health Sciences Simon Fraser University Burnaby British Columbia Canada; ^3^ Division of Health Policy and Management, School of Public Health University of Minnesota Minneapolis Minnesota USA; ^4^ Department of Medicine Emory University School of Medicine Atlanta Georgia USA; ^5^ Department of Population Health Sciences Weill Cornell Medical College New York New York USA; ^6^ Division of Infectious Diseases, Department of Medicine and Center for Dissemination and Implementation, Institute for Public Health Washington University in St. Louis St. Louis Missouri USA; ^7^ Center for AIDS and STD University of Washington Seattle Washington USA; ^8^ Department of Epidemiology University of Washington Seattle Washington USA; ^9^ Division of Allergy and Infectious Diseases Department of Medicine, University of Washington Seattle Washington USA; ^10^ Public Health‐Seattle & King County HIV/STI/HCV Program Seattle Washington USA; ^11^ Department of Epidemiology, School of Public Health Brown University Providence Rhode Island USA; ^12^ Department of Epidemiology Johns Hopkins Bloomberg School of Public Health Baltimore Maryland USA; ^13^ Department of Sociomedical Sciences Columbia University New York New York USA; ^14^ Division of Infectious Diseases and Global Public Health, Department of Medicine University of California San Diego La Jolla California USA; ^15^ Division of Infectious Diseases, Department of Medicine University of Miami Miller School of Medicine Miami Florida USA

**Keywords:** diagnosis, Ending the HIV Epidemic, HIV, implementation science, prevention, RE‐AIM, response, structural interventions, treatment

## Abstract

**Introduction:**

The United States Ending the HIV Epidemic (EHE) initiative was always challenged by structural and implementation barriers to HIV care. On top of that, since 2025, there are additional political and policy barriers that make this goal seem increasingly difficult to achieve. This review identified evidence‐based structural, implementation and biomedical interventions available to support each of the EHE pillars and estimated their potential population‐level impact, accounting for potential barriers.

**Methods:**

We conducted a targeted literature review of US studies presenting data on efficacy or effectiveness of interventions pertaining to the EHE pillars—prevention, diagnosis, treatment and response—published as of September 2025. We searched interventions included in the US Centers for Disease Control and Prevention HIV Compendium and Response Evidence Brief, all publicly available EHE jurisdictional plans, and the peer‐reviewed literature. Potential population impact of an intervention was determined as the product of its effectiveness and scale of delivery. Scale was the product of population reach, provider adoption and structural barriers.

**Results:**

Of 570 studies identified, 57 met the inclusion criteria. We estimated that pre‐exposure prophylaxis (PrEP) uptake could increase as much as nearly two‐fold (risk ratio [RR]: 1.93, 95% CI: 1.30, 1.96) through pharmacies integrated in primary care clinics. Online HIV self‐testing kits were among the most promising interventions to increase testing uptake by two‐fold (RR: 1.99, 95% CI: 1.56, 2.57) Finally, a clinic‐based education app offering tailored health education could result in a 45% absolute increase (RR: 1.45, 95% CI: 1.29, 1.70) in viral suppression among people living with HIV.

**Conclusions:**

Interventions that address barriers to PrEP, HIV testing and adherence to treatment have substantial population‐level impact on HIV care outcomes across EHE pillars through changing the context of how care is offered, understood and delivered.

## 1 Introduction

The United States continues to experience a higher HIV burden than any other industrialized country, despite tremendous advancements in HIV medicine and public health programmes and significant historical investments in the domestic HIV response from 1981 to 2024 [1, 2]. Still, the US Centers for Disease Control and Prevention (CDC) estimates that over 1.2 million people in the United States were living with HIV in 2022, 13% of whom were unaware of their status [3, 4]. HIV is disproportionately concentrated among racial/ethnic, sexual and gender minority communities, and among lower‐income and under‐ or uninsured people [5, 6]. Black/African Americans and Hispanic/Latinx people accounted for 70% of estimated HIV incidence between 2018 and 2022 despite accounting for just 40% of the US population [3].

In 2019, the US government announced the “Ending the HIV Epidemic (EHE)” initiative to reduce HIV incidence by 90% by 2030 through four pillars: (i) early diagnosis of HIV (diagnosis); (ii) effective and rapid treatment for sustained viral suppression (treatment); (iii) HIV prevention of at‐risk individuals (prevention); and (iv) rapid HIV cluster detection and response (response) [7]. Health departments across 57 priority jurisdictions developed plans tailored to each jurisdiction's epidemiological needs by collaborating with local partners [8]. Reaching EHE targets by 2030 is an ambitious goal, and several studies have demonstrated that biomedical interventions alone are inadequate to control the US HIV epidemic or reach EHE targets [5, 6]. Further, the recent cuts to Medicaid will affect access and coverage to HIV prevention and treatment [9, 10].

The drivers of the US HIV epidemic require multi‐level strategies to address the range of barriers limiting access to the highly effective biomedical interventions available for the control of HIV, including pre‐exposure prophylaxis (PrEP) [11, 12]. At an individual level, unstable housing, poverty and other social determinants of health have been associated with a higher risk of poor HIV care outcomes [13]. Barriers to access can be broadly stratified into structural barriers (e.g. incomplete insurance coverage, legal and geographic barriers) [14] and implementation barriers, including the mechanisms of HIV treatment and prevention service delivery, costs, provider training, stigma and mistrust in the healthcare system in the United States [15].

We previously estimated that scaling up evidence‐based biomedical interventions for HIV prevention, diagnosis and treatment in the United States could reduce HIV incidence by up to 50% by 2030 in six US cities, and we concluded that complementary strategies to address barriers in access to care would be required to reach EHE targets [16]. There are a number of evidence‐based interventions that address structural barriers and implementation strategies to promote the adoption, reach and sustainability of evidence‐based practices along the HIV care continuum. There is limited understanding, however, of their potential impact at the population level [16, 17], particularly in settings with diverse demographics, epidemiological conditions and constraints on medical care [18, 19, 20]. Prior systematic reviews have described implementation interventions involving PrEP [21] and HIV testing [22] from studies predominately conducted in low‐ and middle‐income countries, warranting further systematic research in the United States.

This review aimed to identify evidence‐based structural, implementation and biomedical interventions across each of the EHE pillars, and to estimate their potential population‐level impact on HIV care outcomes.

## 2 Methods

### 2.1 Interventions Literature Review

We conducted a targeted literature review to identify evidence‐based structural, implementation and biomedical interventions for each EHE pillar. We searched the following sources: the US Centers for Disease Control and Prevention (CDC) “Compendium of Evidence‐Based and Evidence‐Informed Interventions and Best Practices for HIV Prevention” through the Prevention Research Synthesis Project Publication Search [23], a collection of HIV interventions systematically reviewed dating back to 1996 [24]; the CDC HIV Cluster and Outbreak Detection and Response Evidence Brief [25]; as well as all publicly available jurisdictional EHE plans published as of September 2025. A total of 50 jurisdictional EHE plans were reviewed, representing 55 of 57 EHE priority jurisdictions, excluding Shelby, Tennessee and San Juan, Puerto Rico (details in Supplementary Appendix Table A1).

We included interventions with established efficacy or effectiveness, and which focused on at least one of the EHE pillars of Prevention, Diagnosis, Treatment and Response. Studies were included if they met the following criteria: (i) reports and peer‐reviewed journal articles published between January 2000 and September 2025; (ii) conducted within the United States; (iii) focused on the adult or young adult population (18 years and older); (iv) conducted among participants identified as gay, bisexual and other men who have sex with men (MSM), people who inject drugs (PWID), people living with HIV (PLHIV) or others at risk of HIV acquisition; (v) experimental or observational design; (vi) reported HIV prevention‐, diagnosis‐, treatment‐ or response‐related outcomes; and (vii) published in English.

**FIGURE 1:**
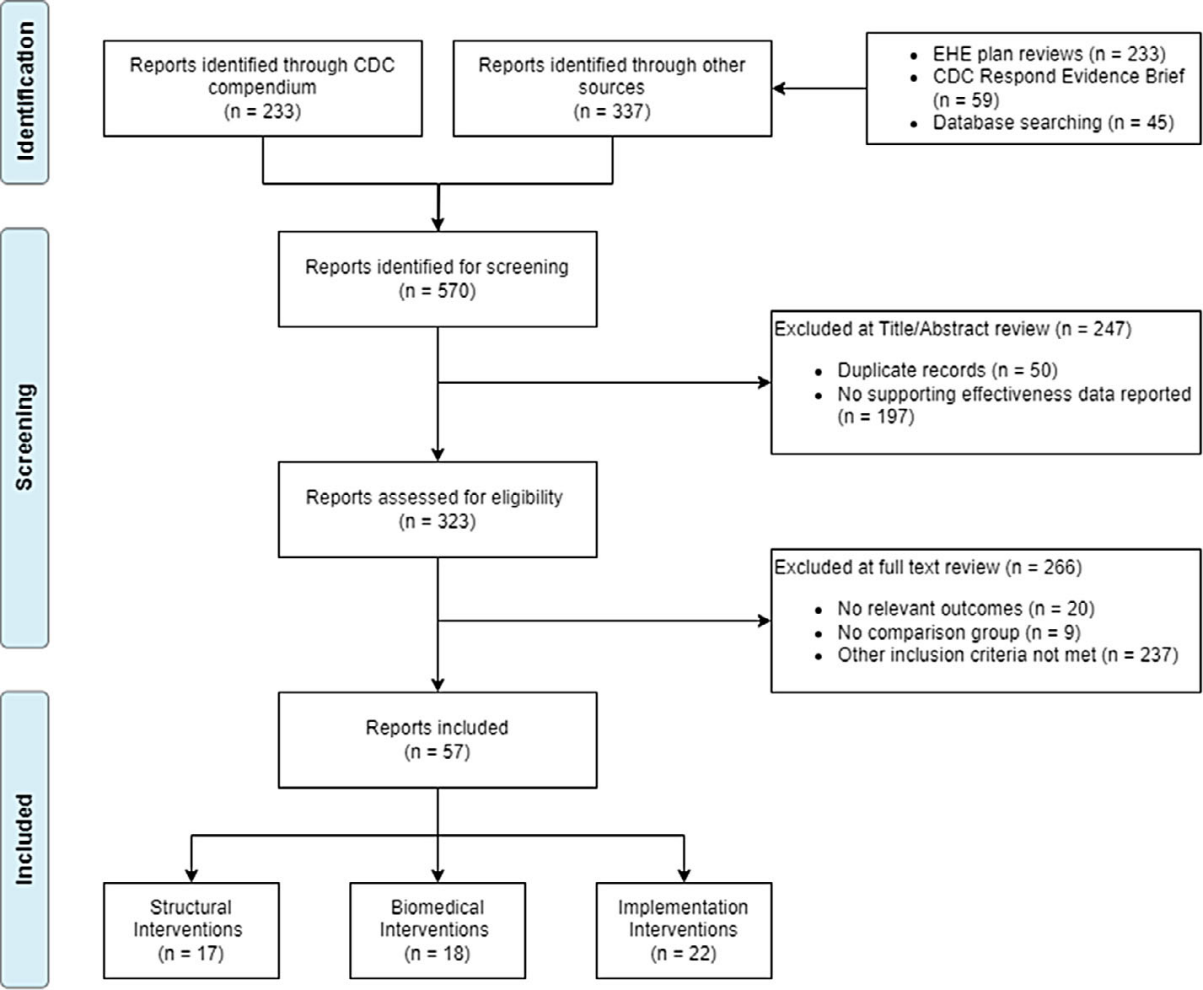
Literature search strategy and intervention selection flow diagram. Abbreviations: CDC, US Centers for Disease Control and Prevention (Compendium of Evidence‐Based and Evidence‐Informed Interventions and Best Practices for HIV Prevention); EHE, Ending the HIV Epidemic (jurisdictional plans).

We excluded studies without reported numerical effect sizes or those without a comparison group. Otherwise, where multiple studies were available for interventions with similar exposures, outcomes and delivery method, we chose the study with the highest quality of evidence (see below). We conducted a manual search of databases including PubMed, MEDLINE, Web of Science and PsycINFO in addition to the grey literature to screen additional articles for interventions with no published experimental evidence of effectiveness (search terms in Supplementary Appendix Table A2). The study selection process is illustrated in Figure 1.

### 2.2 Classifications and Evidence Rating

We categorized interventions according to their orientation on structural‐, implementation‐ or biomedical‐level factors, aligned with classifications in the CDC compendium and implementation science literature [17, 24, 26, 27]. We defined structural interventions as any strategy that entails changes to the environmental, social and economic context in which healthcare is delivered, clinical practice, and/or laws or policy across settings/systems [24] (e.g. Medicaid expansion, healthcare integration, mobile health units, telehealth and HIV case management). Biomedical interventions were defined as the specific treatments, procedures, or other HIV or related services offered within a given setting [27] (e.g. PrEP, syringe service programmes [SSPs], medications for opioid use disorder, HIV testing and rapid antiretroviral therapy [ART]). Implementation interventions were defined as those that seek to improve the reach, acceptance or uptake within a given population of existing evidence‐based interventions across any step of the HIV cascade of care [26] (e.g. physician detailing, provider education and training, patient navigation, clinical decision support tools, mobile apps and health education). We generated the independent effect of the implementation intervention to, for instance increase uptake of a biomedical intervention, which can then be used multiplicatively with base effectiveness levels of the biomedical intervention (i.e. medication type, etc.).

We ranked the quality of evidence for each intervention using the Oxford Centre for Evidence‐based Medicine – Levels of Evidence scale, ranging from level 5 (expert opinion) to level 1a (systematic review of randomized controlled trials [RCTs] and meta‐analyses) [28]. For interventions included in the CDC Compendium, we furthermore distinguished interventions rated as evidence‐based (“rigorously evaluated and provide strongest evidence of efficacy”) or as evidence‐informed (“have some evidence of working and need further testing”) [24]. We otherwise validated the classification definitions and studies selected via a series of three surveys distributed to a scientific advisory committee comprised of US leaders in HIV research. Committee members were additionally asked to provide feedback on the search strategy, the data sources and methodologies utilized, and to provide any additional relevant studies for potential inclusion.

### 2.3 Effectiveness, Scale of Delivery and Population Impact

We estimated the potential population‐level impact of interventions based on the Reach, Effectiveness, Adoption, Implementation, and Maintenance (RE‐AIM) framework for health interventions [29]. For the sake of comparability across interventions with the same primary outcome, we estimated the maximal population‐level impact for the same target population across each intervention type. The target population is defined as a segment of the US population aged 15–64: PrEP uptake/initiation—all MSM, PWID, men who have sex with men and inject drugs, and high‐risk heterosexuals; PrEP adherence—all individuals currently receiving PrEP; HIV testing/HIV positivity rate—all individuals; HIV acquisition (SSP, medications for opioid use disorder)—all PWID and men who have sex with men and inject drugs; ART initiation—all individuals who test positive and are linked to care; ART adherence/drop‐out reduction/retention—all individuals currently on ART; and ART re‐initiation—all ART‐experienced individuals who have dropped out, assuming no constraints in term of reach, adoption and structural barriers. We are not accounting for all the structural constraints, current epidemiological conditions and demographics differences in each EHE jurisdiction.

We presented effectiveness data from each study in the original units of measurement, then standardized outcomes for comparison across interventions on the following outcomes: uptake/initiation of PrEP, adherence to PrEP and reduction in the risk of HIV acquisition (for prevention); the uptake of HIV testing and test positivity rates (for diagnosis); and improvements in ART initiation, adherence and viral suppression rates (for treatment). For interventions focused on the EHE's Response pillar, outcomes were limited to the uptake of prevention, testing or treatment services resulting from partner services, cluster detection and/or HIV outbreak response. We standardized effectiveness estimates (e.g. odds ratio, hazard ratio) reported from each study into relative risk ratio (RR) for comparability [30, 31, 32] (details in Supplementary Appendix Tables A3−A5).

Scale of delivery was defined as the product of reach among service users, adoption within care settings and structural limitations for each intervention *i*, target population *j* and healthcare setting *k*.

Scale*_𝑖j_* = ∑_k_ (reach *_𝑖jk_* x adoption *_𝑖k_* x structural barriers *_𝑖jk_*)

In this application, we estimate scale at the national average; however, these estimates can be generated at the city county or state level. Reach was defined as the participation rate in a given intervention, conditional on the probability that an individual would access and accept the intervention [16, 17]. This included both the eligibility/exclusion criteria for a given intervention (e.g. HIV testing targeted to young, Black MSM, or offered in emergency departments) and the proportion of the eligible group who agreed to receive the intervention, which were primarily estimated from trial‐based response and/or follow‐up rates.

Adoption was defined as the proportion of care settings implementing the intervention [16, 17] or the capacity of providers to deliver an intervention. Factors affecting adoption varied widely across interventions, depending on the resource intensity and logistical challenges of a particular intervention (e.g. intensity of staffing and clinical demands for clinic‐based interventions, vs. interventions that could be delivered via a mobile app).

Structural barriers accounted for financial, geographic, policy or systems‐level barriers restricting intervention access or implementation to a target population (e.g. estimated insurance coverage based on state Medicaid expansion status, estimated PLHIV with adequate geographic access to HIV care, percent of settings with authorization for SSPs) [17]. Structural barriers could potentially affect both reach and adoption; however, the distinct mechanisms of these constraints were disaggregated to articulate their independent effects. Individual‐level income, education and other social determinants of health were not included as structural barriers, as this would decrease comparability across interventions. However, we emphasize the importance of accounting for social determinants of health to maximize an intervention's effect.

Maximal population‐level impact was, therefore, defined as the product of an intervention's effectiveness (i.e. RR) and its scale of delivery (i.e. proportion), assuming that its potential impact is additive to existing service levels (status quo) [16]

$$\begin{array}{ccc} \mathrm{Population}-\mathrm{level}\;\mathrm{impac}t_{ij} & = & \left(\mathrm{effectivenes}s_{ij}\;-\;\mathrm{status}\;\mathrm{qu}o_{ij}\right)\\ & & x\;\mathrm{scal}e_{ij}+\mathrm{status}\;\mathrm{qu}o_{ij} \end{array}$$

For instance, for the PrEP Navigation App, we estimated a potential population‐level impact of 1.37 (pop‐level impact = [effectiveness: 2.19‐1] * [Scale = {Reach = 0.44} * {Adoption = 1} * {structural barriers = 0.76}] + [status quo = 1] = 1.37) or a 37% absolute increase compared to the status quo (no intervention). Detailed information on the definitions, studies included, data sources, calculations and assumptions on conversions to relative risks are provided in Supplementary Appendix Tables A3−A6.

## 3 Results

**FIGURE 2:**
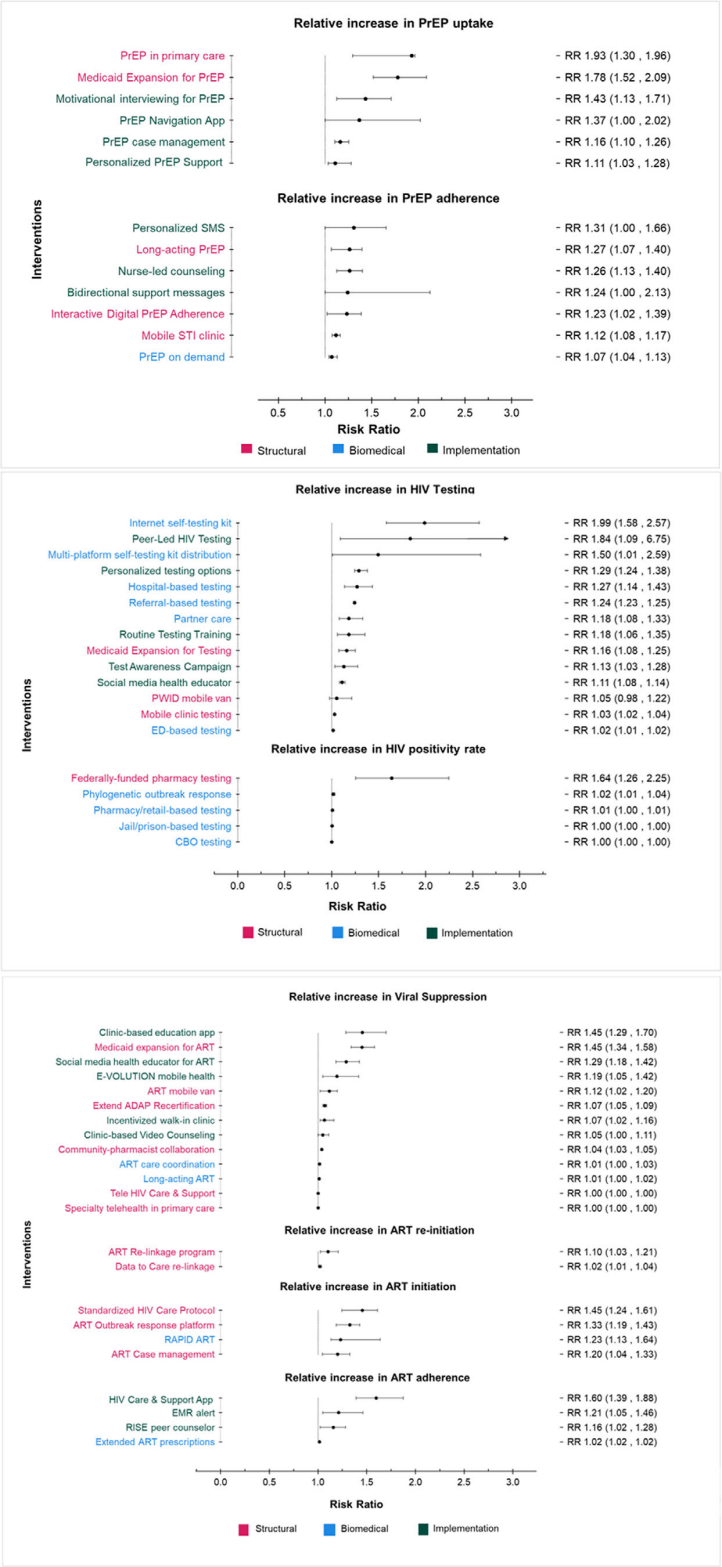
Estimated population‐level impact by effect outcome. (A) PrEP interventions. Refer to Supplementary Appendix Tables A5 and A6 for more details. *The intervention results in a population‐level increase in “Outcome A” by a factor of Y, or a (Y−1)×100 absolute increase compared to the status quo (i.e. no intervention)*. As an example, the intervention—PrEP Navigation App—results in a population‐level increase in PrEP uptake by a factor of 1.37, or a 37% absolute increase compared to the status quo (no intervention). Abbreviations: ADAP, AIDS Drug Assistance Program; ART, antiretroviral treatment; CBO, community‐based organization; CDC, Centers for Disease Control and Prevention; ED, emergency department; EMRs, electronic medical records; PrEP, pre‐exposure prophylaxis; PWUDs, people who use drugs; RAPID, Rapid ART Program for Individuals with an HIV Diagnosis; RR, risk ratio; SMS, short message service (i.e. text message); STI, sexually transmitted infection. (B) HIV testing interventions. Refer to Supplementary Appendix Tables A5 and A6 for more details. *The intervention results in a population‐level increase in “Outcome A” by a factor of Y, or a (Y−1)×100 absolute increase compared to the status quo (i.e. no intervention)*. As an example, the intervention—Internet self‐testing kit—results in a population‐level increase in HIV testing by a factor of 1.99, or a 99% absolute increase compared to the status quo (no intervention). Abbreviations: ADAP, AIDS Drug Assistance Program; ART, antiretroviral treatment; CBO, community‐based organization; CDC, Centers for Disease Control and Prevention; ED, emergency department; EMRs, electronic medical records; PrEP, pre‐exposure prophylaxis; PWUDs, people who use drugs; RAPID, Rapid ART Program for Individuals with an HIV Diagnosis; RR, risk ratio; SMS, short message service (i.e. text message); STI, sexually transmitted infection. (C) ART interventions. Refer to Supplementary Appendix Tables A5 and A6 for more details. *The intervention results in a population‐level increase in “Outcome A” by a factor of Y, or a (Y−1)×100 absolute increase compared to the status quo (i.e. no intervention)*. As an example, the intervention—HIV Care & Support App—results in a population‐level increase in ART adherence by a factor of 1.6, or a 60% absolute increase compared to the status quo (no intervention). Abbreviations: ADAP, AIDS Drug Assistance Program; ART, antiretroviral treatment; CBO, community‐based organization; CDC, Centers for Disease Control and Prevention; ED, emergency department; EMRs, electronic medical records; PrEP, pre‐exposure prophylaxis; PWUDs, people who use drugs; RAPID, Rapid ART Program for Individuals with an HIV Diagnosis; RR, risk ratio; SMS, short message service (i.e. text message); STI, sexually transmitted infection.

A total of 570 reports were identified through the initial search (Figure 1). After removing duplicates and ineligible records at title and abstract screening, 323 reports were further assessed at full‐text review, yielding 57 reports and distinct interventions for inclusion. Of the included studies, 15 interventions focused on HIV prevention, 19 on diagnosis, 23 on treatment, including three under the Response pillar (Table 1; additional details in Supplementary Appendix Table A5). A total of 55 articles (96%) were peer‐reviewed, and two (4%) were drawn from the grey literature, including reports, conference presentations and media articles. Seventeen of the 57 articles (30%) were classified as structural interventions, 18 (32%) as biomedical interventions and 22 (38%) as implementation interventions, as summarized in Table 2. We presented the maximal potential population‐level impact for each intervention in Figure 2 (Panels A−C) with supporting details on the corresponding estimates to derive impact provided in Supplementary Appendix Table A6.

**TABLE 1:** Characteristics of HIV intervention studies included by EHE pillar.

	EHE pillar			
	Prevention^a^	Diagnosis^a^	Treatment^a^	Total *n* (%)
**Studies**	15	19	23	54 (100)
**Classification**				
Structural	3	4	10	17 (31)
Biomedical	4	9	5	18 (33)
Implementation	8	6	8	22 (39)
**Study design**				
Meta‐analysis	2	2	0	4 (7)
RCT	8	6	8	22 (39)
Systematic review	0	1	0	1 (2)
Observational	5	10	15	30 (55)
**Evidence level** ^b^				
1a	2	2	0	4 (7)
1b	8	6	8	22 (39)
2a	0	1	0	1 (2)
2b	2	6	12	19 (35)
2c	3	4	3	10 (19)
**Primary outcomes**				
PrEP uptake	6	—	—	6 (11)
PrEP adherence	7	—	—	7 (13)
HIV acquisition	2	—	—	2 (4)
HIV testing	—	14	—	14 (25)
HIV positivity rate	—	5	—	5 (9)
Viral suppression	—	—	13	14 (24)
ART initiation/re‐initiation	—	—	6	6 (11)
ART adherence	—	—	4	3 (6)

Abbreviations: ART, antiretroviral therapy; CDC, US Centers for Disease Control and Prevention; EHE, Ending the HIV Epidemic initiative; PrEP, pre‐exposure prophylaxis; RCT, randomized controlled trial.

^a^ Includes response interventions (*n* = 3) for HIV prevention (*n* = 0), diagnosis (*n* = 2) and treatment (*n* = 1).

^b^ Trial‐based effectiveness levels of evidence adapted from Oxford Centre for Evidence‐based Medicine—Level 1a—systematic review of RCTs and meta‐analyses; 1b—individual high‐quality RCT; 2a—systematic review of cohort studies; 2b—individual cohort study or quasi‐experimental study; 2c—outcome‐based ecological or multi‐site study (includes secondary analyses). Observational study designs include prospective (*n* = 2), retrospective (*n* = 24), difference‐in‐differences (*n* = 2) and modelling studies (*n* = 1). Percentages may not add up as they are rounded.

**TABLE 2:** Summary of selected interventions by classification and EHE pillar.

Structural	Biomedical	Implementation
**Prevention**		
1. Medicaid expansion for PrEP (2c)	4. Expanded syringe access (1a*)	8. PrEP navigation app (1b*)
2. Mobile STI clinic (2c)	5. Expanded MOUD (1a*)	9. Personalized PrEP support (1b**)
3. PrEP in primary care (2c**)	6. PrEP on demand (2b)	10. PrEP case management (1b)
	7. Long‐acting PrEP (1b)	11. Motivational interviewing for PrEP (1b*)
		12. Interactive digital PrEP adherence (1b*)
		13. Bidirectional support messages (2b**)
		14. Personalized SMS (1b*)
		15. Nurse‐led counselling (1b**)
		
**Diagnosis**		
16. Medicaid expansion for testing (2b)	20. Referral‐based testing (2b)	30. Peer‐led HIV testing (1b)
17. Mobile clinic testing (2b)	21. Hospital‐based testing (2b)	31. Test awareness campaigns (2b)
18. Federally funded pharmacy testing (2c)	22. ED‐based testing (1b*)	32. Social media health educator (1b)
19. PWID mobile van (1b)	23. Jail/prison‐based testing (2a)	33. Routine testing training (2b)
	24. Pharmacy/retail‐based testing (2c)	34. Personalized testing options (1b)
	25. CBO testing (2c)	
	26. Internet self‐testing kits (1b*)	
	27. Multi‐platform self‐testing kit distribution (1a)	
		
		
**Treatment**		
35. Medicaid expansion for ART (2b)	47. ART care coordination (2b*)	50. HIV care and support app (2b**)
36. Extend ADAP recertification (2c)	48. Long‐acting ART (1b)	51. Clinic‐based video counselling (1b*)
37. Tele HIV care and support (2c)	49. RAPID ART (2b*)	52. Clinic‐based education app (2b**)
38. Specialty telehealth in primary care (1b*)		53. Social media health educator for ART (2b**)
39. Standardized HIV care protocol (2b**)		54. EMR alert (1b*)
40. Extend ART prescriptions (2b)		55. Incentivized walk‐in clinic (2b)
41. ART mobile van (1b*)		56. E‐VOLUTION mobile health (2b)
42. Community‐pharmacist collaboration (2b**) 43. ART case management (1b*) 44. ART re‐linkage programme (2b^**^) 45. Data‐to‐care re‐linkage (1b)		57. RISE peer counsellor (1b)
**Response**		
46. ART outbreak response platform (2c)	28. Partner care (1a)	
	29. Phylogenetic outbreak response (2c)	

*Note*: Refer to Supplementary Appendix Table A5 for more details. Trial‐based effectiveness levels of evidence adapted from Oxford Centre for Evidence‐based Medicine—Level 1a—systematic review of RCTs and meta‐analyses; 1b—individual high‐quality RCT; 2a—systematic review of cohort studies; 2b—individual cohort study or quasi‐experimental study; 2c—outcome‐based ecological or multi‐site study (includes secondary analyses); 3a—systematic review of case‐control studies; 3b—individual case‐control study; 4—case studies; 5—expert opinion; Includes if intervention is evidence‐based (*) or evidence‐informed (**) if cited within the CDC Compendium of Evidence‐Based Interventions for HIV Prevention.

Abbreviations: ADAP, AIDS Drug Assistance Program; ART, antiretroviral therapy; CBO, community‐based organization; ED, emergency department; EMR, electronic medical record; HIV, human immunodeficiency virus; MOUD, medication for opioid use disorder; PrEP, pre‐exposure prophylaxis; PWUDs, people who use drugs; RAPID, Rapid ART Program for Individuals with an HIV Diagnosis; SMS, short message service (i.e. text messages); STIs, sexually transmitted infections.

### 3.1 Prevention

Prevention interventions were, by design, designed for PrEP eligible candidates, MSM and young MSM, transgender women, PWID and racial/ethnic minorities at high risk of HIV acquisition, and included two meta‐analyses [33, 34], eight RCTs [35, 36, 37, 38, 39, 40, 41, 42], five observational studies [43, 44, 45, 46, 47] with 43% (*n* = 10) of studies reflecting level I evidence.

PrEP uptake interventions (*n* = 6) ranged in population‐level increase by 11% (RR 1.11 [95% CI: 1.03, 1.28]) in uptake through personalized PrEP support (implementation) to 93% (1.93 [1.30, 1.96]), or nearly a two‐fold increase in uptake through pharmacies and PrEP navigation integrated in primary care clinics (structural) when compared to the status quo, that is no intervention (Figure 2, Panel A) [36, 44]. PrEP adherence interventions (*n* = 7) ranged in population‐level increase by 7% (1.07 [1.04, 1.13]) for PrEP on demand dosing schedules (biomedical) to a 31% increase (1.31 [1.00, 1.66]) in PrEP adherence through personalized daily text messages (implementation) compared to the status quo [41, 46]. Interventions tailored to people who inject drugs aimed to prevent HIV acquisition through shared injections (*n* = 2) and had a population‐level effect of 0.97 (0.95, 0.99) through expanded syringe access programmes (biomedical) and 0.87 (0.84, 0.92) with medications for opioid use disorder (biomedical), resulting in a 3% and 13% absolute risk reduction in HIV acquisition when compared to the status quo, respectively [33, 34].

### 3.2 Diagnosis

Interventions in HIV diagnosis were targeted towards a diverse group of populations, including adults, people with sexually transmitted infections (STIs), those with undiagnosed HIV, MSM, people in correctional facilities, PWID, transgender women, sex workers and clients, partners of PLHIV, racial and sexual minorities, and people accessing specialty care clinics. Cited studies included two meta‐analyses [48], six RCTs [49, 50, 51, 52, 53, 54], one systematic review [55], 10 observational studies [56, 57, 58, 59, 60, 61, 62, 63, 64, 65], with 42% (*n* = 8) reflecting level I evidence.

The population‐level impact of interventions to increase HIV testing uptake (*n* = 14), ranged from a 2% increase (1.02 [1.01, 1.02]) in testing through emergency departments (biomedical) to a nearly doubling in testing (1.99 [1.58, 2.57]) through free internet self‐testing kits (implementation) compared to the status quo (Figure 2, Panel B) [49, 50]. For testing interventions reporting on the HIV positivity rate (*n* = 5), the population‐level impact ranged from a < 1% increase (1.00 [1.00, 1.00]) in HIV detection through community‐based organization testing (biomedical) to a 64% increase (1.64 [1.26, 2.25]) through federally funded HIV testing in pharmacies (structural) compared to the status quo [57, 61].

### 3.3 Treatment

HIV treatment interventions were targeted towards PLHIV including AIDS Drug Assistance Program (ADAP) clients, veterans, young adults and those newly diagnosed, ART‐naive, experiencing ART adherence challenges, as well as racial/ethnic minorities and included eight RCTs [66, 67, 68, 69, 70, 71, 72, 73], and 15 observational studies [74, 75, 76, 77, 78, 79, 80, 81, 82, 83, 84, 85, 86, 87, 88], with 35% (*n* = 8) reflecting level I evidence and 65% (*n* = 15) of studies classified within the CDC compendium as evidence‐based or evidence‐informed.

Among treatment interventions to improve ART initiation (*n* = 4), and compared to the status quo, population‐level increases ranged from 20% (1.20 [1.04, 1.33]) in initiation among ART‐naïve PLHIV through case management (structural), to 45% (1.45 [1.24, 1.61]) in initiation in newly diagnosed PLHIV through standardizing HIV care protocols for non‐HIV specialists to deliver integrated HIV care (structural) (Figure 2, Panel C) [71, 77]. ART re‐initiation (*n* = 2) had a population‐level increase in re‐initiation from 2% (1.02 [1.01, 1.04]) using data‐to‐care re‐linkage between health departments and clinics (structural) to 10% (1.10 [1.03, 1.21]) increase among those lost to care through HIV clinics contacting PLHIV who discontinued ART (structural) [72, 80]. ART adherence interventions (*n* = 4) ranged in population‐level increase of 2% (1.02 [1.02, 1.02]) in ART adherence through extending ART prescription lengths to 90 days (structural) to 60% (1.60 [1.39, 1.86]) through an ART adherence support app for young adults (implementation) [78, 84]. For ART interventions reporting on viral suppression (*n* = 13), the population‐level increase ranged by <1% (1.00 [1.00, 1.00]) for HIV care offered through specialty telehealth in primary care (structural) to 45% (1.45 [1.29, 1.70]) through a clinic‐based smartphone app offering tailored information for HIV, health education and stress reduction (implementation) [66, 85].

### 3.4 Response

Interventions included in this pillar were targeted towards people at risk of HIV due to an outbreak or partners becoming newly diagnosed with HIV, including PWID, and included one meta‐analysis [89] and two observational studies [62, 81], with only partner care [89] reflecting level I evidence.

Two of three interventions included under the Response pillar involved outbreak response for diagnosis (implementation) [62] and treatment (structural) [81] (Figure 2, Panels A−C). The population‐level impact ranged from 2% (1.02 [1.01, 1.04]) for phylogenetic outbreak response to increase the HIV positivity rate in HIV testing (implementation) [62] to 33% (1.33 [1.19, 1.43]) for ART initiation through an outbreak response platform (structural) [81], compared to the status quo. Lastly, partner care (biomedical) [89] could increase HIV testing uptake by 18% (1.18 [1.08, 1.33]) compared to the status quo.

## 4 Discussion

This review identified interventions for each EHE pillar and estimated the maximal potential impact on key outcomes if scaled to the population‐level in the United States. Interventions with the greatest potential population impact could result in nearly a two‐fold increase in PrEP uptake with pharmacies integrated in primary care clinics (structural), as well as nearly doubling HIV testing uptake and viral suppression through free internet self‐testing kit distribution (implementation) and through clinic‐based mobile apps that enhance HIV health education and disease management (implementation), respectively. Though we have considered these interventions individually, no single intervention will be sufficient in an epidemic response, and factors such as community need/desire, feasibility and cost‐effectiveness may all be factors in local decision‐making. Further, our results are generated entirely from studies executed in the United States, thus representing interventions motivated by gaps in care, and unmet healthcare needs of this focal population. Other developed‐ and developing‐world settings may have disparate needs and health system capabilities that may expand on the listing of interventions generated; we believe the exercise is informative and replicable in other settings.

We found that many interventions with a high degree of efficacy in trial settings had a smaller potential for population‐level impact, as they were constrained by either a focused target population, limited acceptability among recipients, diminished capacity for adoption among providers and other structural factors affecting accessibility or implementation (e.g. insurance coverage). While ART has been available to PLHIV who are uninsured and underinsured through the Ryan White HIV/AIDS Program, costs of PrEP and HIV testing that are not covered by insurance pose constraints to access and uptake, highlighting the need for increased PrEP affordability with examples being restriction of cost sharing for labs and follow‐up visits, generic PrEP options, drug assistance programmes, as well as subsidized HIV testing [90, 91, 92]. Medicaid expansion is the most effective strategy to increase PrEP affordability; however, 10 states have yet to fully expand Medicaid coverage under the Affordable Care Act, and 20 (35%) of the 57 EHE priority jurisdictions are in states that have not adopted Medicaid expansion at the time of writing [93]. This has resulted in higher proportions of MSM who are insured in non‐expansion states (72% vs. 88% in expansion states) [43], which substantially reduced the population impact for most of the PrEP and testing interventions. Several included studies reported that Medicaid expansion in these settings could increase PrEP uptake by 1.78‐fold [43], HIV testing by 1.16‐fold [65] and viral suppression among ADAP recipients by 1.45‐fold increase [74]. Dramatic reductions in federal public health funding [94] have left the fate of the EHE initiative uncertain, as the proposed funding for fiscal year 2026 would drastically reduce the budget for HIV prevention through the CDC, as well as treatment through Ryan White, both of which are critical to EHE [95]. Additionally, the recently signed Federal Budget Reconciliation Bill [96] significantly cuts Medicaid funding by $1 trillion dollars [97] and will be unlikely that states that have not already expanded Medicaid would be willing to do so [98]. This will undoubtedly have implications for the course of the HIV epidemic in the United States. While SSPs have demonstrated effectiveness for reducing HIV transmission risk through sharing of injection drug equipment [34], the scalability of this intervention is constrained by state authorization of SSPs. To date, 12 states do not explicitly or implicitly authorize SSPs, and nine (16%) of the EHE priority jurisdictions are located in states without authorization [99].

HIV self‐testing strategies were estimated to have the highest potential impact on testing rates among those reviewed. Distribution of free, home‐based HIV self‐testing kits through peer‐led outreach in social media [51] could result in a nearly two‐fold increase in HIV testing at the population level, with high acceptance. Two other self‐testing studies [48, 50]—one of which was a national trial [50]—involved distribution strategies through web‐based promotion and ordering and reported similarly high rates of testing uptake among MSM. Four studies involved HIV tests offered at no cost and in low‐barrier approaches through mobile clinics [54, 56], federally funded testing in retail pharmacies [57] and mobile apps [51]. HIV testing may be offered at no cost for those without insurance in community‐based settings and with no co‐pay or deductible for people with private or public insurance [100]. As the reported mean cost of HIV testing in STI clinics is approximately $22 [101], $42 in retail pharmacies [57] and $60 USD for online self‐testing kits [102, 103], the out‐of‐pocket cost for self‐testing may remain prohibitive for marginalized communities. While HIV testing is a key pillar of the EHE strategy, its accessibility remains greatly constrained, particularly within clinical settings in the United States [16, 92]. Despite CDC recommendations since 2006 that advised everyone between the ages of 13−64 to undergo HIV screening with opt‐out testing at least once as part of routine medical care [104], in a national study of HIV care providers in the United States, only 60% of providers reported offering HIV screening to patients 13−64 years of age [105]. The proposed budget cuts for fiscal year 2026 further decimate the HIV prevention infrastructure that the CDC provides by funding state and local health departments [106, 107], the effects of which have been felt in Florida [108] and Georgia [109]. Furthermore, over half (*n* = 29) of the 57 EHE priority jurisdictions are in states that criminalize HIV. HIV and STI criminalization statutes also pose concerns for the EHE's Response pillar efforts to understand transmission patterns and identify outbreaks, highlighting a key area for HIV legal reforms [110].

Among treatment interventions reporting on viral suppression, we estimated that the highest impact intervention could increase viral suppression by 45% through a clinic‐based mobile app with tailored educational resources for HIV health management and stress reduction [85] due to its high levels of acceptance and scalability. Long‐acting injectable ART has shown promise in increasing viral suppression among PLHIV with ART adherence challenges [68]; however, population impact may be minimal, as only 5% of national estimates of Ryan White HIV/AIDS Program Part C clinics have procedures and policies in place to implement long‐acting ART programmes [111]. Given that long‐acting ART is accessed through Ryan White and ADAP, its availability varies between states [112], and its US Food and Drug Administration's approval is aimed towards people who are virally suppressed [113], the population that stands to benefit the least from this biomedical intervention. However, with the recent approval for lenacapavir as long‐acting PrEP [114] and current efforts to expand capability to provide long‐acting ART in Ryan White clinics [115], the limited scalability of these interventions may be more promising at a local level if viral suppression is a requirement to access long‐acting ART.

Of the three interventions included under the response pillar [62, 81, 89], the population‐level impact of outbreak response interventions had the largest increase for ART initiation by 1.33‐fold through agency collaboration to reduce time to initial HIV care for PLHIV newly diagnosed [81]. Assisted partner notification (where providers contact partners of newly diagnosed PLHIV and offer testing) could result in an 18% increase in population‐level HIV testing uptake compared to passive referral, as reported in an included meta‐analysis [89]. This represents a valuable strategy for expansion in the United States and has been implemented in some settings, including hospitals across NYC neighbourhoods hardest hit by the HIV epidemic [116]. Partner services are a key component of the Response pillar; however, national data indicate that among CDC‐funded tests conducted in 2021, only 50% of PLHIV newly diagnosed were offered a partner services interview (excluding missing/invalid records) [117]. Additionally, a survey of EHE state and local jurisdictions indicated that fewer than 50% of contacted partners of newly diagnosed PLHIV had an HIV test from partner services [118]. We found that these measures of scale would thus reduce the impact of assisted partner notification on HIV testing uptake. Even though public health efforts to increase routine HIV testing must also support strategies to enhance partner services and linkage to care [119, 120], it is exceedingly difficult in the resource‐constrained public health environment and is further limited by the current administration's proposed budget cuts.

### 4.1 Barriers to Population‐Level Implementation

The majority of barriers to interventions’ reach were related to restrictions in the target population, which limited the potential scope due to the setting where they are implemented, such as hospitals [49, 59], jails/prisons [55] or Veteran Affairs clinics [66, 78], compared to Medicaid, which has a greater reach at a population‐level [43, 65]. We also saw wide variability in acceptance rates, which were derived from trial‐based estimates of participation and follow‐up, as well as larger‐scale studies such as the percentage of individuals who would take a free HIV test if offered at a pharmacy [121]. Structural barriers were primarily a result of limitations in insurance coverage, as we assumed that financial barriers and out‐of‐pocket expenditures would inherently limit the population‐level impact, particularly for more discretionary interventions such as testing and PrEP. In a recent decision, the US Supreme Court upheld PrEP coverage under the Affordable Care Act, requiring most insurers to cover preventive services rated “A” or “B” by the US Preventive Services Task Force [122]. There were varying estimates of insurance coverage across the target populations for different interventions; however, there was a clear distinction in coverage among MSM in states that had adopted Medicaid expansion (87.9%), versus those that had not (71.6%) [43]. We also noted a recent study that reported upwards of 84% of those indicated for PrEP have insurance coverage [123]; however, the recent eligibility changes to Medicaid enacted by the 2025 Federal Budget Reconciliation Bill [124] will undoubtedly increase rates of uninsurance across the country. Barriers to adoption were the most difficult to quantify, as there was a paucity of data for provider‐side limitations, which are context‐specific. Capacity‐building initiatives for HIV service providers were cited in 24 EHE jurisdictional plans; and while national initiatives such as the CDC's Capacity Building Assistance Provider Network [125] serves the nation's HIV prevention workforce through training, technical assistance and other supports for CDC‐funded health departments, and community‐based organizations, there is currently no published programme data.

These findings extend our prior work, which established a framework to estimate the population impact of evidence‐based biomedical [16] and implementation [17] interventions in HIV care across six US cities experiencing a high burden of HIV. It is important to note that our results are meant to highlight the potential limitations in the scalability of these interventions at a national level, due to intervention‐specific and external factors, and not the epidemiological impact or cost‐effectiveness. Interventions may have substantial, focused epidemiological impact if they are implemented in jurisdictions with more concentrated HIV epidemics among their target population (e.g. an HIV testing intervention tailored to young, Black MSM in Atlanta was found to be more cost‐effective than in other jurisdictions and populations) [17]. Similarly, there would likely be greater variability in cost‐effectiveness depending on the implementation components of each intervention. For example, once developed, app‐based interventions could be scaled at relatively low cost compared to clinic‐based, personnel‐intensive interventions.

### 4.2 Limitations

This study had several limitations. First, the search was conducted only in English. Despite searching multiple databases and other literature sources not previously considered, it is possible some studies were missed due to evolving terminologies for the interventions listed. Second, there was a lack of standardization in reported outcome measures for several interventions—specifically for uptake or adherence in PrEP and ART—with some PrEP studies measuring clinic visits, time to medication pick up, and proportion of medication days covered and some ART studies measuring time to relinkage, time to treatment initiation and treatment adherence at variable time points (3, 6 or 12 months). For studies with variable time points, we chose the highest effect sizes. Finally, structural and implementation barriers considered for each intervention were not exhaustive and may act on other contextual barriers to scalability not considered; however, the data collected reflect the best available programme‐specific national‐level data to inform these estimates. Though we are presenting a single set of effect sizes, these may be different by setting depending on demographics, epidemiological conditions and constraints on medical care. Moreover, the review focused only on studies executed in the United States. While the scope of available interventions will be comparable in most developed‐world settings, their results are generated within a unique population and epidemiological conditions; estimates of population impact will necessarily vary in international settings. Despite these limitations, this study represents the first to extensively review interventions for each EHE pillar and to systematically quantify their potential population‐level impact while accounting for individual‐ and structural‐level determinants of care engagement. Future work will evaluate how differences in population‐level scale and targeted interventions translate into epidemiological impact and cost‐effectiveness for EHE jurisdictions.

## 5 Conclusions

We estimated that some structural and implementation HIV interventions had high potential for population‐level scalability. Policy‐ and systems‐level changes in service provision can address inequities among populations experiencing the greatest burdens of HIV. Implementation interventions, including the use of mobile health modalities, can enhance service delivery for care providers and for the people whose services are intended to reach. Our results demonstrate the breadth of evidence‐based structural, implementation and biomedical interventions currently available across all EHE pillars and quantify the potential reach, adoption and structural barriers to achieving scale. Addressing these barriers with structural and implementation interventions, in addition to biomedical interventions, is critical for service providers and policymakers to maximize the public health impact of advancements in HIV care.

## Author Contributions

MP, BCG‐A, MTV, BE, and BN developed the manuscript and contributed to each section. MP, BCG‐A and MGM conducted the literature review. MP, MTV, BCG‐A, and BE analysed and interpreted the data. XZ, WSA, CB, CdR, EE, EHG, MRG, BDLM, SHM, LRM, BRS, SAS, and HET reviewed and edited multiple iterations of the manuscript. All authors approved the final manuscript.

## Funding

This study was supported by the National Institutes on Drug Abuse (R01DA041747; PI: Nosyk). The funding agreement ensured the authors' independence in designing the study, interpreting the data, writing and publishing the report.

## Role of the Funding Source

The funding source was independent of the design of this study and did not have any role during its execution, analyses, interpretation of the data, writing or decision to submit results. All authors had full access to the results in the study and take responsibility for the integrity of the data and accuracy of analysis.

## Conflicts of Interest

The authors declared no conflicts of interest.

## Supporting information




**Supporting File 1**: jia270146‐sup‐0001‐SuppMat.docx

## Data Availability

The data that support the findings of this study are available in the Supplementary Material of this article.
