# Supplementary material for: What Tools Do We Have to End the US HIV Epidemic? A Review of Structural, Biomedical and Implementation Interventions and Their Potential Population Impact
Source: J Int AIDS Soc. 2026 Jul 9;29(7):e70146. doi: 10.1002/jia2.70146 (PMC13351116; doi:10.1002/jia2.70146)
Supplement: Supplementary file 1 — Supporting File 1: jia270146‐sup‐0001‐SuppMat.docx [file JIA2-29-e70146-s001.docx]

**Supplementary Appendix**

**What tools do we have to end the US HIV epidemic?** **A review of structural, biomedical and implementation interventions and their potential population impact**

Micah Piske, Brenda Carolina Guerra-Alejos, Minh Tri Van, Benjamin Enns, Melanie G Medina, Xiao Zang, Wendy S Armstrong, Czarina Behrends, Carlos del Rio, Eva Enns, Elvin H Geng, Matthew R Golden, Brandon DL Marshall, Shruti H Mehta, Lisa R Metsch, Bruce R Schackman, Steffanie A Strathdee, Hansel E Tookes, Bohdan Nosyk, on behalf of the Localized Economic Modeling group

**Table A1.** Ending the HIV Epidemic (EHE) jurisdiction plans included in literature review

| **Jurisdiction (census region, county, state)** | **Title of EHE Plan** | **Year** |
| --- | --- | --- |
| ***Midwest*** |  |  |
| Cook, IL | Ending the HIV Epidemic Plan for Cook County 2021-2025 | 2020 |
| Marion, IN | Marion County, Indiana Ending the HIV Epidemic (EHE) Plan 2021-2025 | 2020 |
| Cuyahoga, OH | Plan to END THE HIV EPIDEMIC: Cuyahoga County, Ohio | 2020 |
| Franklin, OH | Plan to END THE HIV EPIDEMIC: Central Ohio (Franklin County) | 2020 |
| Hamilton, OH | Plan to END THE HIV EPIDEMIC: Hamilton County, Ohio | 2020 |
| Missouri, MO | Ending the HIV Epidemic: A Plan for Missouri | 2021 |
| Wayne, MI | Ending the HIV Epidemic in Wayne County 2020– 2025 Strategic Plan | 2020 |
| ***Northeast*** |  |  |
| Essex, NJ | Ending the HIV Epidemic: New Jersey’s Strategic Plan: Essex and Hudson Counties 2020-2030 | 2020 |
| Hudson, NJ |  |  |
| Bronx, NY | New York City 2020: Ending the HIV Epidemic: A Plan for America Plan | 2020 |
| Kings, NY |  |  |
| New York City, NY |  |  |
| Queens, NY |  |  |
| Philadelphia, PA | A Community Plan to End the HIV Epidemic in Philadelphia | 2020 |
| Suffolk, MA | Ending the HIV Epidemic in Suffolk County Massachusetts | 2020 |
| ***South*** |  |  |
| Broward, FL | Florida’s Unified Ending the HIV Epidemic | 2020 |
| Duval, FL |  |  |
| Hillsborough, FL |  |  |
| Orange, FL |  |  |
| Palm Beach, FL |  |  |
| Pinellas, FL |  |  |
| Miami-Dade, FL | Florida’s Unified Ending the HIV Epidemic  ENDING THE HIV EPIDEMIC IN MIAMI-DADE COUNTY Accelerating Local and State HIV Planning to End the Epidemic | 2020 |
| Cobb, GA | ENDING THE HIV EPIDEMIC IN GEORGIA | 2020 |
| DeKalb, GA |  |  |
| Fulton, GA |  |  |
| Gwinnett, GA |  |  |
| East Baton Rouge Parish, LA | East Baton Rouge Parish Ending the HIV Epidemic Plan 2020-2025 | 2020 |
| Orleans Parish, LA | Orleans Parish Ending the HIV Epidemic Plan | 2020 |
| Baltimore, MD | ENDING THE HIV EPIDEMIC Baltimore City Draft Plan 2020-2030 | 2020 |
| Montgomery, MD | A Plan to End HIV in Montgomery County | 2020 |
| Prince George's County, MD | The Prince George’s County Ending the HIV Epidemic Plan | 2020 |
| Mecklenburg, NC | Ending the HIV Epidemic Plan (EHE) – Mecklenburg County, NC | 2020 |
| Bexar, TX | Texas Response to Federal Ending the HIV Epidemic Phase One Jurisdictions Planning | 2020 |
| Dallas, TX |  |  |
| Tarrant, TX |  |  |
| Travis, TX |  |  |
| Harris, TX* | Houston Health Department CDC-RFA-PS19-1906 – Strategic Partnerships and Planning to Support Ending the HIV Epidemic in the United States Component B: Accelerating Local and State HIV Planning to End the HIV Epidemic | 2020 |
| Washington, DC | Ending the HIV Epidemic in the District of Columbia for All Communities by 2030 | 2020 |
| Alabama | Ending the HIV Epidemic Alabama | 2020 |
| Arkansas | Ending the HIV Epidemic in Arkansas 2020-2025 Strategic Plan | 2020 |
| Kentucky | Kentucky Ending the HIV Epidemic Strategic Plan 2021 – 2026 | 2020 |
| Mississippi | Mississippi’s Ending the HIV Epidemic Plan | 2021 |
| Oklahoma | Ending the HIV Epidemic: A Plan for Oklahoma | 2020 |
| South Carolina | ENDING THE HIV EPIDEMIC PLAN South Carolina | 2020 |
| Maricopa, AZ | Ending the HIV Epidemic in Maricopa County, AZ | 2020 |
| Alameda, CA | Alameda County CALIFORNIA CONSORTIUM FOR CDC PS19-1906 | 2021 |
| Los Angeles, CA | Ending the HIV Epidemic in Los Angeles County | 2020 |
| Orange, CA | Orange County CALIFORNIA CONSORTIUM FOR CDC PS19-1906 | 2021 |
| Riverside, CA | Riverside County CALIFORNIA CONSORTIUM FOR CDC PS19-1906 | 2021 |
| Sacramento, CA | Sacramento County CALIFORNIA CONSORTIUM FOR CDC PS19-1906 | 2021 |
| San Bernardino, CA | San Bernardino County CALIFORNIA CONSORTIUM FOR CDC PS19-1906 | 2021 |
| San Diego, CA | San Diego County CALIFORNIA CONSORTIUM FOR CDC PS19-1906 | 2021 |
| San Francisco, CA | Ending the Epidemics: COLLECTIVE STRATEGIES FOR ADDRESSING HIV, HEPATITIS C, AND SEXUALLY TRANSMITTED INFECTIONS IN SAN FRANCISCO | 2020 |
| Clark, NV | State of Nevada Ending the HIV Epidemic (EHE) Plan | 2021 |
| King, WA | Plan to Support Ending the HIV Epidemic in King County | 2020 |

Plans reviewed as of August 2023. 50 plans reviewed representing 55 of 57 Ending the HIV Epidemic Phase 1 priority jurisdictions, (excluding Shelby County, Tennessee and San Juan Puerto Rico). Georgia plan currently unavailable online. * Harris County, TX initial EHE plan submitted on December 31st, 2020 reviewed.

**Table A2. Search terms used for database manual search**

| mobile clinic[All Fields] AND "pre exposure prophylaxis"[All Fields] |
| --- |
| "Affordable Care Act"[All Fields] AND "pre exposure prophylaxis"[All Fields] |
| (("sexual health"[MeSH Terms]) AND ("clinic"[title/abstract])) AND ("HIV prevention"[Title/Abstract]) |
| (("ADAP"[All Fields]) OR "drug assistance programs"[All Fields])) AND ("HIV"[All Fields]) AND ("renewal"[All Fields]) |
| (("academic detailing"[Title/Abstract]) OR ("academic training"[Title/Abstract])) AND ("pre exposure prophylaxis"[MeSH Terms]) |
| ("pre exposure prophylaxis"[MeSH Terms]) AND ("patient navigation"[MeSH Terms]) |
| educ*[All Fields] AND "social media"[All Fields] AND "viral suppression"[All Fields] |

**Table A3. Definitions for effectiveness, reach, adoption, structural barriers, and population-level impact**

| **Category** | **Definition** | **Range** | **Interpretation** |
| --- | --- | --- | --- |
| **Effectiveness** | Intervention-derived measures of effectiveness (e.g. odds ratios, risk ratios, hazard ratios). For this comparison, we standardized these estimates into relative risks (RR) compared to the status quo using assumptions described below   Note: A value of **1** indicates **no impact**, while values **greater than 1** indicate a **positive impact**. All measures have been converted to reflect positive outcomes.  For interventions without reported uncertainty estimates for outcomes (e.g. studies reporting only proportions between comparison groups), we derived 95% confidence intervals utilizing the Clopper-Pearson interval exact binomial test for proportions (116)(3) and non-parametric tests (117)(4) for studies reporting only the median effect sizes. | 0 to infinity | Participants who received intervention A are X.XX times as likely to have "outcome A" compared to those who did not receive the intervention. Participants who received intervention A are (X.XX-1) % as likely to have "outcome A" compared to those who did not receive the intervention. |
| **Reach** | **Individual-level access and acceptability for a given intervention**: i) probability the individual will access services (eligibility or exclusion criteria which limit the intervention to a subset of the population) and/or ii) the probability that the individual will accept the intervention. | 0-1 | X.XX% of participants  **either met eligibility criteria and accessed, or accepted** intervention A |
| **Adoption** | **Provider-level capacity:** The capacity of providers to deliver the intervention if scaled to a population level. | 0-1 | X.XX% of providers are equipped to offer this intervention A at scale. |
| **Structural barriers** | **Factors that are external to providers or patients:** structural factors such as those related to financial, geographic, legal/policy barriers limiting intervention implementation and access within the target population. | 0-1 | X.XX% of participants can receive intervention A when accounting for structural barriers unrelated to the individual, provider, or the intervention itself. |
| **Population-level impact** | Estimates the population-level effectiveness of the intervention among its target population, adjusting for reach, provider adoption, and structural limitations, using the formula(Effectiveness -1)* Reach * Adoption * Structural limit +1 | 0 to infinity | The intervention results in a population-level increase in 'outcome A' by a factor of Y or an (Y-1)*100% absolute increase compared to the status quo |

**Table A4. Conversion assumptions**

| **Measure/s** | **Conversion to RR or equivalent (reference)** | **Assumptions** |
| --- | --- | --- |
| Relative Risk (RR), Risk Ratio, or Rate Ratio | N/A | Rate Ratio assumed equivalence in ratio to risk ratio between exposed and unexposed groups |
| Hazard Ratio (HR) | RR=HR* | * Proportional hazard assumption |
| Odds Ratio (OR) | RR = OR / (1 – p_0_ + (p_0_ * OR))  where p is the risk in the control/status quo group (1) | None |
| Absolute Risk (reduction), Risk Difference (RD), Any % difference in outcome reported | RR=p_1_/(p_1_-RD) where p_1_ is the risk (range 1-0) in the intervention group* | * Where p_1_ is not known, assumption that p_1_=100% |
| Proportion* | RR= [Risk of outcome (incidence proportion) in intervention group] / [Risk of outcome (incidence proportion) in control group] | N/A |
| Relative Risk reduction (RRR) | RR=1-RRR/100% | N/A |

*Risk Ratio= (A/A+B) / (C/C+D) for determining RR from proportion of participants with outcome by intervention exposure (2).

For ease of comparison, we reframe reductions in negative outcomes as improvements in positive outcomes. For example, if a study reports that an intervention reduces the likelihood of dropping out of ART, we interpret this as an increase in ART retention by taking the inverse of the reported value.

**Table A5.** Characteristics of intervention studies and details on effectiveness

| **Intervention ID** | **Intervention, (Classification) [reference]** | **Intervention Description** | **Location (Study N)** | **Setting** | **Focal Population** | **Study design, Evidence Level (**BI or EI if in CDC compendium)* | **Effectiveness** | **Effect Outcome** | **Effect Description** | **Effectiveness (RR) (95% CI)** |
| --- | --- | --- | --- | --- | --- | --- | --- | --- | --- | --- |
| ***Prevention & Response - Pre-exposure prophylaxis (PrEP) interventions*** | | | | | | | | | | |
| 1 | Medicaid expansion for PrEP (Structural) [Baugher, 2021, (3)] | PrEP coverage and utilization in Medicaid expansion vs. non-expansion states. | 23 US cities (n = 8857) | NHBS survey data | MSM | Cross- sectional, 2c (-) | 1.78 (1.52, 2.09) | PrEP uptake | MSM living in expansion states were 1.78 times as likely to use PrEP (crude prevalence ratio 1.78 [95% CI 1.52, 2 .09]). | 1.78 (1.52, 2.09) (no conversion) |
| 2 | Mobile STI clinic (Structural) [Doblecki-Lewis, 2024,(4)] | Mobile clinic offering package of barrier-lowering strategies for mobile HIV /STI prevention and treatment. | Miami-Dade County, FL (n=1896 clients) | Multi-site mobile clinic, and fixed clinic | Priority locations identified by HIV incidence | Observational cohort study, 2c (-) | 1.68 (1.44,1.96) | PrEP adherence | PrEP persistence among people initiating PrEP in mobile clinics was higher compared to those initiating in fixed location aHR 1.68 (1.44,1.96) (at 48 weeks). | 1.68 (1.44, 1.96) (no conversion) |
| 3 | PrEP in primary care  (Structural) [Coleman, 2020, (5)] | Integrates routine medical care with a peer navigator tool providing insurance navigation and an on-site pharmacy. | Washington, DC (n=198) | 2 FQHC | PrEP-eligible candidates | Cross-sectional pre-post, 2c (EI) | 0.45 (0.45,0.72) | PrEP uptake | Post-intervention arm experienced a significantly shorter mean time to PrEP medication pickup vs. pre-intervention (1.19 mean days [SD = 2.42] vs. 2.61 mean days [SD = 5.74], t(146) = 2.19, p = 0.030). 55% decrease | 2.19 (1.38, 2.23) (RD to RR) |
| 4 | Expanded Syringe Access (Biomedical) [Aspinall, 2014 (6)] | Expanded access to clean injection equipment for the prevention of parenteral HIV transmission for people who inject drugs (PWID). | USA (N=3 studies), Other (N=3 studies) | SSPs | PWID | Meta-analysis, 1a (EB) | 0.42 (0.22, 0.81) | HIV acquisition | The pooled effect estimate of risk of HIV among PWID exposed to needle and syringe programs was 0.42 (0.22, 0.81). | 0.42 (0.22, 0.81) (no conversion) |
| 5 | Expanded MOUD (Biomedical) [MacArthur, 2012 (7)] | Expanded access to office-based medication for opioid use disorder with buprenorphine for PWID | USA (N=4), Other (N=5) | Primary care and opioid treatment program | PWID | Meta-analysis, 1a (EB) | 0.46 (0.32, 0.67) | HIV acquisition | Opiate substitution was associated with a 54% reduction in the risk of HIV infection (rate ratio 0.46, 95% CI 0.32 to 0.67). | 0.46 (0.32, 0.67) (no conversion) |
| 6 | PrEP on demand (Biomedical [Stansfield, 2023 (8)] | Likelihood of adhering to daily PrEP through daily or on-demand 2-1-1 PrEP strategies. | Harlem, NY (n=179), Bangkok, Thailand (n=178) | Community clinic and clinical research site | MSM | Agent-based stochastic model based on HPTN 067, 2b (-) | 1.18 (1.11,1.28) | PrEP adherence | Among MSM for whom on-demand PrEP was optimal, mean effectiveness improved by 18% compared to a daily regimen. | 1.22 (1.12, 1.39) (RD to RR) |
| 7 | Long-acting PrEP (Biomedical) [Landovitz, 2021 (9)] | Comparison of long-acting injectable cabotegravir for HIV prevention given every 8 weeks to daily oral tenofovir disoproxil fumarate-emtricitabine (TDF-FTC). | 7 countries including USA (n=4566) | Hospitals and outpatient clinics | At-risk MSM and transgender women who have sex with men | RCT, 1b (-) | 91.5% | PrEP adherence | 72.3% (65.4%,85.7%) of samples had tenofovir–diphosphate levels consistent with taking at least four TDF–FTC doses per week in the prior 1–2 months (control), while 91.5% of person-years were covered by injectable CAB-LA placebo with injection delays of less than 2 weeks. | 1.27 (1.07,1.40) (RR: % intervention/ %control) |
| 8 | PrEP Navigation App (Implementation) [Sullivan, 2022, (10)] | App that delivers tailored prevention messaging including PrEP eligibility screener and service locators for PrEP care. | Atlanta, GA; Detroit, MI; New York City, NY (n=1226) | Multi-site (venues, social media) | GBMSM and other MSM | RCT, 1b (EB) | 2.41 (1.00,5.76) | PrEP uptake | Odds of using PrEP in 3 months after intervention compared to control adjusted: aOR 2.41 (1.00 - 5.76) | 2.19 (1.00, 4.32) (OR to RR) |
| 9 | Personalized PrEP Support (Implementation) [Desrosiers, 2019, (11)] | Culturally tailored counseling intervention with personalized PrEP counseling session with a staff member who identifies as a black MSM. | Washington, DC (n=50) | Outpatient Clinics | YBMSM | RCT, 1b (EI) | 0.24 (0.09,0.45) | PrEP uptake | A greater proportion of intervention participants reported initiating PrEP in the last 3 months compared to control (24%] vs. 0 [0%]; p =.023). | 1.32 (1.10, 1.82) (RD to RR) |
| 10 | PrEP case management (Implementation) [Doblecki-Lewis, 2019, (12)] | Strengths-based case management and PrEP navigation by trained staff providing information on PrEP, available resources, and support. | Miami, FL (n=61) | Outpatient clinics and CBOs | PrEP-eligible candidates | RCT, 1b (-) | 1.38 (1.24,1.59) | PrEP uptake | Forty percent of people with PrEP navigation started PrEP in less than 12 weeks compared to 29% in control group. | 1.38 (1.24, 1.59) (RR: % intervention/ %control) |
| 11 | Motivational interviewing for PrEP (Implementation) [Chan, 2021, (13)] | Brief behavioral intervention for PrEP uptake with motivational interviewing to determine PrEP suitability during STI/HIV testing. | USA, unspecified (n=87) | STI clinic | MSM with HIV behavioral risk factors | RCT, 1b (EB) | 3.6 (1.5-8.9) | PrEP uptake | Intervention participants were significantly more likely to receive and accept a prescription for PrEP compared to control participants (OR = 3.6; 95% CI: 1.5 to 8.9). | 2.09 (1.32, 2.78) (OR to RR) |
| 12 | Interactive Digital PrEP Adherence (Implementation) [Liu, 2019, (14)] | Multicomponent mHealth intervention with SMS and youth-tailored interactive online content to enhance PrEP adherence. | Chicago, IL (n=121) | STI clinic | At-risk YMSM | RCT, 1b (EB) | 2.06 (1.07,3.99) | PrEP adherence | Odds of visits with protective TFV-DP levels among participants compared to standard of care participants after adjustment for baseline differences in depression. aOR 2.06 (1.07 -3.99) | 1.28 (1.03, 1.48) (OR to RR) |
| 13 | Bidirectional support messages (Implementation) [Fuchs, 2018, (15)] | Weekly bidirectional text or e-mail support messages to encourage PrEP adherence. | San Francisco, Chicago, USA (n=56) | Multi-site | MSM | Pre-post, 2b (EI) | 0.284 (0.2,0.646) | PrEP adherence | Pre-post analysis showed a 28% increase (95% CI 0.2-64.6; p = 0.048) in the medication possession ratio (measure of adherence) when comparing visits just before and after entering iText. | 1.40 (1.00, 2.80) (RD to RR) |
| 14 | Personalized SMS (Implementation) [Moore, 2018, (16)] | Personalized text messaging systems for adherence promotion. | California (n=398) | Multi-site (4 medical centers) | MSM and transgender women | RCT, 1b (EB) | 1.56 (1.00,2.42) | PrEP adherence | iTAB arm was more adherent for the composite adherence outcome aOR 1.56 (1.00–2.42) Adjusted for age. | 1.37 (1.00, 1.79) (OR to RR) |
| 15 | Nurse-led counseling (Implementation) [Mayer, 2017, (17)] | Nurse-delivered, individual-level behavioral intervention for PrEP adherence, sexual behaviour and barriers to adherence. | Boston, MA (n=50) | Primary care clinic for sexual and gender minority patients) | High-risk MSM | RCT, 1b (EI) | 1.33 (1.16,1.50) | PrEP adherence | Eighty-four percent of intervention participants had drug levels consistent with daily TDF/FTC compared to 63% of comparison participants at 6 months (p = 0.03). | 1.33 (1.16, 1.50) (RR: % intervention/ %control) |
| ***Diagnosis & Response - HIV testing interventions*** | | | | | | | | | | |
| 16 | Medicaid Expansion for testing (Structural) [Farkhad, 2021, (18)] | Increased insurance coverage and access to health care to increase identification of undiagnosed HIV infection and use of HIV prevention. | USA (N not provided, state-level estimates) | CDC national data | General | Cross-sectional difference-in-differences, 2b (-) | 1.139 (1.07, 1.20) | HIV testing | Medicaid expansions were associated with a significant increase in HIV diagnoses a 13.9% (0.508 × 100/3.659) increase from pre-expansion levels. | 1.16 (1.08, 1.25) (RD to RR) |
| 17 | Mobile clinic testing (Structural) [Ellen, 2003, (19)] | Comparing characteristics between clients seen aboard a mobile STD/HIV clinic to those seen in a traditional municipal STI/HIV clinic. | Baltimore City, MD (n=5909) | Multi-site (mobile and standard health clinics) | People with STDs, PLHIV | Retrospective cohort, 2b (-) | 1.12 (1.09,1.15) | HIV testing | 504 (72.0%) mobile van clients accepted testing for HIV, compared to 353 (64.4%) of municipal clinic clients. | 1.12 (1.09, 1.15) (RR: % intervention/ %control) |
| 18 | Federally-funded pharmacy testing (Structural) [Collins, 2018, (20)] | State health department funded retail pharmacies (Walgreens) to provide HIV testing in areas with large racial/ethnic minority communities and high rates of poverty. | Virginia, USA (n=3630) | 32 retail pharmacies | General | Cross- sectional, 2c (-) | 2.00 (1.40, 2.95) | HIV positivity rate | 0.8% tests reactive to HIV (sample size=3630) compared to 0.4% positivity in clinical settings. | 2.00 (1.40, 2.95) (RR: % intervention/ %control) |
| 19 | PWID mobile van (Structural) [Page KR, 2024,(21)] | PWID-focused integrated care van (ICV) visited neighborhood sites weekly and offered a broad range of PWID-focused services in a welcoming atmosphere | Baltimore (n = 720) | integrated care van versus care as usual (clinic) | PWID | Cluster-randomized trial, 1b | 4.37 (0.80, 23.8) | HIV testing | Compared with usual services, participants in the ICV arm were more likely to have had an HIV test in the past 6 months (OR = 4.37). | 1.20 (0.90, 1.73) (OR to RR**,** where p0 =78.6%) |
| 20 | Referral-based testing (Biomedical) [Katz, 2016, (22)] | Health Departments modified STI partner services programs to provide partner services to all MSM with STIs and ensure those without a prior HIV diagnosis tested for infection. | Washington State (n=8133) | State-wide | MSM with early STI infection | Pre-post, 2b (-) | 1.44 (1.42,1.47) | HIV testing | Among MSM without a prior HIV diagnosis, HIV testing among PS recipients increased from 63% to 91% (p < 0.001). | 1.44 (1.42, 1.47) (RR: % intervention/ %control) |
| 21 | Hospital-based testing (Biomedical) [Felsen, 2017, (23)] | Hospital-based EMR test alert. The prompt appeared for patients with no prior HIV test or a high-risk diagnosis after their last HIV test. | Bronx, NY (n=36,610) | 3 hospitals | General | Pre-post, 2b (-) | 2.78 (2.62, 2.96) | HIV testing | While the alert was active, HIV testing increased among total admissions aOR: 2.78 (2.62, 2.96). | 2.38 (2.27, 2.50) (OR to RR) |
| 22 | ED-based testing (Biomedical) [Montoy, 2016, (24)] | Individuals visiting a hospital emergency room (ER) receive a routine HIV test unless they decline. | San Francisco, CA (n=4800) | Hospital | General | RCT, 1b (EB) | 1.28 (1.24, 1.31) | HIV testing | Compared to opt-in testing, the was an absolute difference of 27.9% (24.4%-31.3%) of HIV testing percentage in the opt-out arm. | 1.39 (1.32, 1.45) (RD to RR) |
| 23 | Jail/prison-based testing (Biomedical) [Iroh, 2015, (25)] | Randomized, routine, opt-out and opt-in testing in jails. | USA & Canada (n=9418,60 studies) | Jails and prisons | People in correctional facilities | Systematic review, 2a (-) | 0.01674 (0.0166,0.0169) | HIV positivity rate | The proportion of positive tests from opt-in and opt-out testing in jails and prisons was 1.7% (47263/2,822,856 tests). | 1.02 (1.0169, 1.0172) (RD to RR) |
| 24 | Pharmacy/Retail-based testing (Biomedical) [Weidle, 2014, (26)] | Point-of-care HIV testing at pharmacies and retail clinics | USA (n=1540) | 21 sites (pharmacies and clinics) | General | Retrospective (pilot) study, 2c (-) | 0.016 (0.010,0.023) | HIV positivity rate | Out of 1540 tests conducted, 24 (1.6%) had reactive test results. | 1.02 (1.01,1.02) (RD to RR) |
| 25 | CBO testing (Biomedical) [Sanchez, 2014, (27)] | FOCUS program uses clinical and community-based partnerships for non-clinical settings for HIV testing | 10 USA cities (n=799,573 tests) | Hospitals, outpatient clinics, community health centers, community partners | General | Retrospective cohort, 2c (-) | 0.0094 (0.009,0.010) | HIV positivity rate | Out of 101,239 tests conducted in community settings, 948 had a positive test (0.94%). | 1.009 (1.009, 1.010) (RD to RR) |
| 26 | Internet self-testing kit (Biomedical) [MacGowan, 2020, (28)] | HIV self-test kits distribution via internet. Participants mailed two self-test kits with counselor access (phone) and a link to HIV prevention information and local testing locations. | USA (n=2665) | Home-based | MSM | RCT, 1b (EB) | 2.38 (1.90, 2.98) | HIV testing | The cumulative number of newly identified infections during the trial was twice as high in ST participants as control participants (25 of 1325 [1.9%] vs 11 of 1340 [0.8%]). | 2.38 (1.90, 2.98) (RR: % intervention/ %control) |
| 27 | Multi-platform self-testing kit distribution (Biomedical) [Eshun-Wilson, 2021, (29)] | HIV self-testing distribution strategies including online ordering and mail distribution, facility and community distribution. | North America, S Africa, Asia and the Pacific (N=7 US studies) | Multi-site (home-based and health facility) | MSM, TGW, clients of FSW | Meta-analysis, 1a (-) | 1.55 (1.01,2.76) | HIV testing | RR 1.55, (95% CrI: 1.01–2.76) uptake of web-based HIV self-testing kits compared to traditional facility based (US, Asia, and Pacific studies). | 1.55 (1.01, 2.76) (no conversion) |
| 28 | Partner care (Biomedical) [Dalal, 2017, (30)] | Assisted partner notifications for HIV test uptake and diagnosis and occurrence of adverse events to inform the development of normative guidelines. | 8 countries including USA (n=5150 index cases, 6127 partners) | Outpatient clinics, hospitals, health departments | Partners of PLHIV | Meta-analysis, 1a (-) | 1.46 (1.22,1.75) | HIV testing | Assisted partner notification services resulted in an increase in HIV testing uptake among partners compared with passive referral (RR = 1.46; 95% CI: 1.22-1.75). | 1.46 (1.22, 1.75) (no conversion) |
| 29 | Phylogenetic outbreak response (Implementation) [McClung, 2021, (31)] | Phylogenetic analysis of HIV surveillance data and polymerase sequences, as well as federal, state and local agencies collaboration for HIV testing, PrEP and SSP expansion. | West Virginia, USA (n=82) | Community | PWID | Retrospective cohort, 2c (-) | 0.03 (0.01,0.06) | HIV positivity rate | DIS interviewed 49 index cases from outbreak and initiated follow-up with 303 partners and 225 contacts of index cases and partners: 9 HIV diagnoses were identified from 303 tests (3.0% positivity). | 1.03 (1.01, 1.06) (RD to RR) |
| 30 | Peer-Led HIV Testing (Implementation) [Young, 2013, (32)] | Peer leaders deliver information about HIV via Facebook groups and participants may request a free, home-based HIV testing kit. | Los Angeles, CA (n=112) | Social networking sites | MSM (African American or Hispanic/Latinx) | RCT, 1b (-) | 2.0 (1.11,7.88) | HIV testing | Nine of 25 intervention participants (36%) who requested the test took it and mailed it back compared with 2 of 11 control participants (18%). | 2.00 (1.11, 7.88) (RR: % intervention/ %control) |
| 31 | Test Awareness Campaign (Implementation) [Habarta, 2017, (33)] | Increase testing rates through communication campaigns by local and international channels (including online, mobile and magazine ads) that encourage HIV testing. | 8 USA cities (n=702) | Online survey data | Black gay, bisexual and other MSM | Pre-post, propensity score matching, 2b (-) | 1.38 (1.11,1.73) | HIV testing | Those who reported exposure to TMUS had 1.38 (95% CI [1.11, 1.73]) times the probability of reporting they got tested for HIV in past 6 months compared to those not reporting exposure. | 1.38 (1.11, 1.73) (no conversion) |
| 32 | Social media health educator (Implementation) [Rhodes, 2016, (34)] | Health educator facilitated online content on social media frequented by MSM to promote testing, local resources and private Q&As. | USA, unspecified (n=1292) | Social media sites | MSM | RCT, 1b (-) | 1.52 (1.44,1.60) | HIV testing | 64% participants tested for HIV in past 12m post-intervention vs. 42% control group. | 1.52 (1.44, 1.60) (RR: % intervention/ %control) |
| 33 | Routine Testing Training (Implementation) [Lubelchek, 2013, (35)] | Training sessions for non-HIV specialist providers to increase routine HIV testing in specialty care. | Chicago, IL (n=5543) | Specialty clinics | Non-HIV specialist care providers | Cross-sectional, 2b (-) | 2.57 (1.51, 4.36) | HIV testing | OR: 2.57 (1.51,4.36) testing post-intervention period - largest difference across clinics. | 2.06 (1.40, 2.86) (OR to RR) |
| 34 | Personalized testing options (Implementation) [Frye, 2020, (36)] | Brief computerized baseline assessment for receipt of personalized HIV testing recommendations and information. | NYC, NY(n=236) | Online survey | YBMSM and TGW who have sex with men or TGW | RCT, 1b (-) | 1.85 (2.02,1.80) | HIV testing | 41% participants tested within 3m at baseline vs. 76% tested within 3m post intervention. | 1.85 (1.80, 2.02) (RR: % intervention/ %control) |
| ***Treatment & Response – ART interventions*** | | | | | | | | | | |
| 35 | Medicaid Expansion for ART (Structural) [Furl, 2018, (37)] | Expansion of Medicaid to ADAP recipients. | Nebraska, USA (n=284) | Single site (hospital) | PLHIV | Retrospective cohort, 2b (-) | 1.45 (1.34,1.58) | Viral suppression | 84.8% of insured ADAP program participants had undetectable viral load compared to 58.4% of uninsured ADAP recipients. | 1.45 (1.34, 1.58) (RR: % intervention/ %control) |
| 36 | ADAP Recertification Support (Structural) [Erly, 2023, (38)] | A large proportion of clients in Washington state fail to recertify during ADAP enrollment maintenance and are disenrolled from care impacting medication discontinuation. | Washington state (n=5480) | State-wide | ADAP clients | Cross-sectional, 2c (-) | 1.12 (1.09,1.15) | Viral suppression | Of the 1336 ADAP clients who disenrolled ≥1 time, 83% were virally suppressed before disenrollment versus 69% after (RD 12%, 95%CI 9–15%). | 1.14 (1.10, 1.18) (RD to RR) |
| 37 | Tele HIV Care & Support (Structural) [Salgado, 2021, (39)] | A comprehensive HIV telehealth system, including telemedicine to provide remote care through clients’ local medical homes. A telementoring program was also initiated to strengthen the HIV provider workforce. | Georgia (n=6977) | State-wide | PLHIV | Retrospective cohort, 2c (-) | 1.04 (1.03,1.05) | Viral suppression | 951/1,041 (91.4%) Ryan White Part B clients receiving telehealth services were virally suppressed compared to 6,126/6,977 (87,8%) all Georgia RWHAP Part B clients. | 1.04 (1.03, 1.05) (RR: % intervention/ %control) |
| 38 | Specialty telehealth in primary care (Structural) [Ohl, 2019, (40)] | Improve viral suppression among clients through offering specialty care via telehealth in nearby VA primary care clinics instead of distant HIV clinics located within VA facilities. | USA, unspecified (n=1670) | Multi-site (primary care clinics) | PLHIV at VA clinics | RCT, 1b (EB) | 1.14 (1.01,1.3) | Viral suppression | Telehealth patients had greater improvement in documented viral suppression during the evaluation year compared to control patients (91.5% vs 80.0%, respectively; RR, 1.14; 95% CI, 1.01 - 1.30). | 1.14 (1.01, 1.30) (no conversion) |
| 39 | Standardized HIV Care Protocol (Structural) [Halperin, 2018, (41)] | Agency-wide operation procedure including training sessions for providers to standardize workflow for non-HIV specialists to deliver same care, immediate ART and HIV health/social services. | New Orleans, LA (n=71) | Single site (FQHC) | Newly diagnosed PLHIV | Cross-sectional , 2b (EI) | 0.45 (0.33, 0.50) | ART initiation | The intervention group had a reduced median time to viral suppression (<200 copies/mm3) compared to the historical cohort (30 days vs. 68 days, [95% CI: 60 - 92]). | 1.82 (1.49, 2.00) (RD to RR) (Translated to increase in ART initiation) |
| 40 | Extended ART prescriptions (Structural) [McGinnis, 2021, (42)] | Extending ART prescriptions ≥90 days through a mail delivery service of ART. | USA (n=27,674) | National | Veterans with HIV | Retrospective cohort, 2b (-) | 1.0526 (1.0519,1.0534) | ART adherence | No impact of COVID-19 on ART coverage from 2019 to 2020 (76% to 80%). | 1.053 (1.052, 1.053) (RR: % intervention/ %control) |
| 41 | ART mobile van (Structural) [Altice, 2007, (43)] | Improve adherence to ART and reduce viral load through direct administration of ART with community health care mobile clinics and appointment reminders. | New Haven, CT (n=141) | Mobile health van | PWID LHIV | RCT, 1b (EB) | 2.6 (1.2, 5.5) | Viral suppression | At 6 months post-initiation of intervention, a greater proportion of intervention participants achieved virologic success than comparison participants (70.5% vs. 54.7%; OR = 2.6, 95% CI = 1.2 to 5.5,). | 1.39 (1.08, 1.59) (OR to RR) |
| 42 | Community-pharmacist collaboration (Structural) [Byrd, 2020, (44)] | Improving adherence through integration of community-based pharmacists with HIV medical providers to develop therapy-related action plans. | 10 US cities (n=765) | Outpatient clinics/ pharmacies | PLHIV on ART | Retrospective cohort, 2b (EI) | 1.23 (1.21,1.25) | Viral suppression | Sustained viral suppression in the post vs. pre-implementation period (80% vs. 65%, p < 0.001). | 1.23 (1.21, 1.25) (RR: % intervention/ %control) |
| 43 | ART case management (Structural) [Gardner, 2005, (45)] | HIV clinics offer the intervention to ART-naïve PLHIV to increase probability of ART initiation. The intervention included up to five contacts with a case manager over a 90-day period. | 4 US cities (n=1838) | HIV clinics | ART-Naive PLHIV | RCT, 1b (EB) | 1.41 (1.10, 1.60) | ART initiation | Patients with case-management were more likely to visit an HIV clinician compared to standard of care RR 1.41 (1.10, 1.60). | 1.41 (1.10, 1.60) (no conversion) |
| 44 | ART Re-linkage program (Structural) [Bove, 2015, (46)] | HIV clinics implement the program and successfully contact a proportion who have dropped out of ART to increase the probability of ART re-initiation. | Seattle, WA (n=753) | HIV clinic | PLHIV not on ART | Prospective cohort with controls, 2b (EI) | 1.70 (1.20, 2.30) | ART re-initiation | Time to relinkage was shorter on patients in the intervention group compared to historical controls (aHR 1.70 (1.20, 2.30)). | 1.70 (1.20, 2.30) (no conversion) |
| 45 | Data to Care re-linkage (Structural) [Fanfair, 2021, (47)] | Collaborative data-to-care strategy between health departments and clinics to identify people newly out of HIV care aiming to increase re-engagement, viral suppression, and retention in care. | CT, MA, Philadelphia (n=1893) | Multi-site (clinics, health centers, FQHCs, Ryan White clinics) | PLHIV not on ART | RCT, 1b | 54.9% (51.2%-61.2%) | ART re-initiation | Comparing the intervention to standard of care, re-engagement outcomes were: All sites 525 (54.9%) vs 394 (42.1%) (*p* < 0.0001); CT 170 (51.2%) vs 135 (41.9%) (*p* = 0.02); MA, 167 (52.7%) vs 138 (44.1%) (*p* = 0.03); PHL, 189 (61.2%) vs 121 (40.3%) (*p* < 0.0001) | 1.23 (1.21, 1.25) (RR: % intervention/ %control) |
| 46 | ART Outbreak response platform (Structural) [End Stigma, End HIV Alliance, 2020, (48)] | Full investigation of an HIV outbreak led to a collaboration among patient advocates, city public health officials and academic staff to create a platform to link people to providers for immediate care. | San Antonio, TX (n=4 agencies, 23 clients) | Multi-site | Young gay and bisexual Hispanic/Latino men who have sex with men | Retrospective, 2c (-) | 1.63 (1.40, 1.75) | ART initiation | Median time between HIV diagnosis and initial care appointment fell from 13 days to 2 days. | 1.63 (1.40, 1.75) (RR: % intervention/ %control – assuming 30 days is 0%, -57% vs. 93%) |
| 47 | ART care coordination (Biomedical) [Robertson, 2018, (49)] | HIV clinics offer the intervention (which combines various evidence-based elements into a package) to eligible PLHIV currently receiving ART to reduce the probability of ART drop-out. | NYC, NY (n=90,361) | Registry data | PLHIV on ART | Retrospective cohort, 2b (EB) | 1.10 (1.07, 1.13) | Viral suppression | People enrolled in care coordination were 1.1 times as likely to have viral load suppression compared to non-enrollees RR 1.10 (1.07, 1.13). | 1.10 (1.07, 1.13) (no conversion) |
| 48 | Long-acting ART (Biomedical) [Rana, 2024, (50)] | Phase III trial comparing LAI vs. oral standard of care (SOC) ART in PWH with a history of suboptimal adherence (persistent HIV-1 RNA >200 c/mL or loss to follow-up). | US (n=293) | Multi-site (Health centers/ hospitals) | PLHIV with ART adherence challenges | RCT, 1b (-) | 0.182 (0.311. 0.054) | Viral suppression | Long-acting ART recipients had reduced virologic failure (7.2%) compared to those receiving standard of care ART (25%) [-18.2% difference nominal 31.1%, 5.4%]. | 1.22 (1.06, 1.45) (RD to RR) |
| 49 | RAPID ART (Biomedical) [Pilcher, 2017, (51)] | HIV clinics offer the intervention for same-day ART initiation to newly diagnosed individuals to increase probability of immediate ART initiation. | San Francisco, CA (n=86) | Single site (Ward-86 HIV clinic) | Newly diagnosed PLHIV | Retrospective cohort, 2b (EB) | 1.32 (1.23, 1.54) | ART initiation | Percentage difference in ART uptake at 30 days (31.9%) between individuals receiving intervention (100%) and those not receiving RAPID ART (68.1%). | 1.47 (1.30, 2.17) (RD to RR) |
| 50 | HIV Care & Support App (Implementation) [Zurlo, 2020, (52)] | Improve retention in care and viral suppression through mobile app for reminders, virtual health visits, managing goals, secure messaging, lab results, and general HIV information. | Harrisburg, PA (n=92) | Multi-site (4 health centers/ outpatient clinics) | Young adults with HIV | Pre-post, 2b (EI) | 1.90 (1.66,2.20) | ART adherence | A greater percentage of participants were retained in care in post-intervention period at 6 months compared to baseline (78.6% vs. 41.3%, respectively). | 1.90 (1.66, 2.20) (RR: % intervention/ %control) |
| 51 | Clinic-based Video Counseling (Implementation) [Lewis, 2022, (53)] | Web-based video doctor intervention delivered to PLHIV while in the clinic with tailored content about medication initiation and adherence, sexual risk reduction, and other behaviors to decrease HIV transmission risk. | USA (n=799) | 4 HIV primary care clinics | PLHIV | RCT, 1b (EB) | 1.14 (1.00-1.29) | Viral suppression | Male participants receiving Positive Health Check were more likely to achieve suppression at 12 months than male participants receiving standard of care adjusted risk ratio [aRR] [95% CI = 1.14 (1.00 to 1.29)]. | 1.14 (1.00, 1.29) (no conversion) |
| 52 | Clinic-based education app (Implementation) [Dillingham, 2018, (54)] | Clinic-based smartphone app with tailored educational resources including clinic orientation, information on HIV and health, and stress reduction techniques. | Virginia, US (n=77) | Ryan White clinic | PLHIV | Prospective cohort, 2b (EI) | 1.85 (1.60,2.19) | Viral suppression | Participants achieved viral suppression at 6 (87% vs. 47%, p < 0.001) months post intervention compared to baseline. | 1.85 (1.60, 2.19) (RR: % intervention/ %control) |
| 53 | Social media health educator for ART (Implementation) [Tanner, 2018, (55)] | Improve retention in HIV care, increase viral suppression through social media individual-level intervention with cyberhealth educators using messaging through social media platforms tailored to participant context and needs. | Guildford county, NC (n=91) | Social media | Racially/ethnically diverse YMSM and TGW LHIV | Pre-post, 2b (EI) | 1.45 (1.32,1.60) | Viral suppression | Participants who achieved viral suppression increased from 61.3% at pre-enrollment to 88.8% at the end of the 12-month implementation period. | 1.45 (1.32, 1.60) (RR: % intervention/ %control) |
| 54 | EMR alert (Implementation) [Robbins, 2012, (56)] | Clinical decision support tool for alerts in EMR to notify providers of suboptimal patient follow up and test outcomes. | Boston, MA (n=1011) | Hospital HIV clinic | PLHIV on ART | RCT, 1b (EB) | 0.69 (0.53, 0.90) | ART adherence | The rate of 6-month suboptimal follow-up was lower in the intervention group compared to the control group (IRR: 0.69 (0.53, 0.90)). | 1.45 (1.11, 1.89) (Translated to increase in ART retention) |
| 55 | Incentivized walk-in clinic (Implementation) [Dombrowski, 2019, (57) | Walk-in primary care clinic with intensive case management support and incentives for achieving viral suppression and completing visits | Seattle, WA (n=150) | Outpatient clinic | PLHIV poorly engaged in HIV care | Pre-post, 2b | 3.2 (1.8–5.9) | Viral suppression | Max Clinic patients had more improvement in viral suppression than control patients (aRRR, 3.2; 95% confidence interval [CI], 1.8–5.9) | 3.2 (1.8–5.9)  (no conversion) |
| 56 | E-VOLUTION mobile health (mHealth) intervention with a human support element. (Implementation) [Gerke, D. R., 2023, (58)] | Using text messaging to improve HIV care outcomes among youth and young adults living with HIV. | St Louis, MO (n=100) | HIV clinic sites at Washington University School of Medicine | Youth and young adults with HIV | Pre-post, 2b (EI) | 1.43 (1.12, 1.84) | Viral suppression | Results of frequencies also demonstrated that of the 74 participants who completed 12 months of the intervention, 32 remained virally suppressed, 20 became virally suppressed, 14 remained virally unsuppressed, and 8 became virally unsuppressed during the full intervention period. Compare to At baseline, no. of. Viral suppression : 49/ 100 (no. total) | 1.43 (1.12-1.84) |
| 57 | RISE peer counselor (structural) [Bogart 2022,(59)] | Trained peer counselor to conduct motivational interviewing (MI) with Black or African American PWH to address self-identified barriers to taking ART. | Los Angeles County, CA (n=166) | Community-based HIV services organization | Black or African American persons with HIV (PWH) on antiretroviral therapy (ART) but not yet virally suppressed and/or non-adherent to ART | RCT, 1b (EB) | 2.00 (1.10-3.60) | ART adherence | A greater number of participants in the intervention than the control condition showed optimal (at least 75%) adherence at nearly every time-point except baseline. The repeated-measures logistic regression indicated a significant intervention effect on dichotomous adherence, OR (95% CI) = 2.0 (1.1–3.6), p = 0.03 | 1.28 (1.04-1.46) (OR to RR**,** where p0 =56.1%) |
| Abbreviations: RD: Risk difference; RR: relative risk; PrEP: pre-exposure prophylaxis; ART: antiretroviral treatment; NHBS: National HIV Behavioral Surveillance; EMR: electronic medical record; CBO: community-based organization; TDF/FTC: Tenofovir disoproxil fumarate/emtricitabine; DIS: Disease intervention specialist; TMUS: Testing makes us stronger; YBMSM: young black men who have sex with men; MSM: men who have sex with men; aHR: adjusted hazard ratio; PWID: people who inject drugs; FQHC: federal qualified health center; SHC: sexual health clinic; ADAP: AIDS drug assistance program; CDC: Centers for Disease Control and Prevention; DID: Differences-in-differences; STI: Sexually transmitted infection; IDU: injection drug use; PLHIV: people living with HIV; VA: veteran affairs; HPTN: HIV Prevention Trials Network; ACTG: Advancing Clinical Therapeutics Globally for HIV/AIDS and Other Infections; TGW: transgender women; FSW: female sex workers; IRR: incidence rate ratio; SSP: syringe service program; RAPID: Rapid ART Program for Individuals with an HIV Diagnosis; RCT: randomized controlled trial; RWHAP: Ryan White HIV/AIDS Program; aRRR: adjusted relative risk ratio. | | | | | | | | | | |
| *Trial-based effectiveness levels of evidence adapted from Oxford Centre for Evidence-based Medicine – Level 1a – systematic review of RCTs and meta-analyses; 1b – individual high-quality RCT; 2a – systematic review of cohort studies; 2b – individual cohort study or quasi-experimental study; 2c - outcome-based ecological or multi-site study (includes secondary analyses); 3a – systematic review of case-control studies; 3b – individual case-control study; 4 – case studies; 5 – expert opinion; Includes if intervention was evidence-based (EB) or evidence-informed (EI) if cited within the CDC Compendium of Evidence-Based Interventions for HIV Prevention. | | | | | | | | | | |

**Table A6.** Intervention effectiveness, reach, adoption and scalability for estimating population-level impact

| **Intervention ID** | **Intervention (Classification) [reference]** | **Effectiveness (RR) (95% CI)** | **Primary outcome** | **Reach** | **Adoption** | **Structural barriers** | **Population-level impact (RR) (95% CI)*** |
| --- | --- | --- | --- | --- | --- | --- | --- |
| ***Prevention & Response - Pre-exposure prophylaxis (PrEP) interventions*** | | | | | | | |
| 1 | Medicaid expansion for PrEP (Structural) [Baugher, 2021, (3)] | 1.78 (1.52, 2.09) | PrEP uptake | N/A | N/A | N/A | 1.78 (1.52 ,2.09) |
| 2 | Mobile STI clinic (Structural) [Doblecki-Lewis, 2024, (4)] | 1.68 (1.44, 1.96) | PrEP adherence | 46% (507/1109 mobile clinic patients in this study sought PrEP services) | 45% of mobile health units receive federal funding(60) | 84% of people indicated for PrEP in the US have insurance coverage(61) | 1.12 (1.08 ,1.17) |
| 3 | PrEP in primary care  (Structural) [Coleman, 2020, (5)] | 2.19 (1.38, 2.23) | PrEP uptake | N/A | 93% of US health centers have dedicated staff for HIV outreach and PrEP services(62) | 84% of people indicated for PrEP in the US have insurance coverage(61) | 1.93 (1.30 ,1.96) |
| 4 | Expanded Syringe Access (Biomedical) [(Aspinall, 2014 (6)] | 0.42 (0.22, 0.81) | HIV acquisition | 18% of PWID will use SSPs every month(63) | 42.4% of US health centers have dedicated staff for PWID services(64) | 76% (38/50 states allow SSP authorization in the US)(65) | 0.97 (0.95 ,0.99) |
| 5 | Expanded MOUD (Biomedical) [[MacArthur, 2012 (7)] | 0.46 (0.32, 0.67) | HIV acquisition | 66% of people will accept MOUD ( Inverse of estimate: 100%-34% = 66% assumes to not have problems filling their MOUD prescription) (66) | 37.5% of qualified clinician offer buprenorphine prescription(67) | 95% of Affordable Care Act marketplace plans covered all forms of MOUD(68) | 0.87 (0.84 ,0.92) |
| 6 | PrEP on demand (Biomedical [(Stansfield, 2023 (8)] | 1.22 (1.12, 1.39) | PrEP adherence | 36% (On-demand PrEP was optimal for 36% of MSM participants in this study) | 93% of US health centers have dedicated staff for HIV outreach and PrEP services(62) | Financial barrier not applied as population is already on PrEP | 1.07 (1.04 ,1.13) |
| 7 | Long-acting PrEP (Biomedical) (Landovitz, 2021 (9)] | 1.27 (1.07, 1.40) | PrEP adherence | 100% Assume people on PrEP will accept long-acting PrEP and have optimal adherence | 100% Assume most providers who already prescribe PrEP can prescribe long-acting injectable PrEP | 100% insurance covered long-acting PrEP(69). Financial barrier not applied because population is already on PrEP | 1.27 (1.07 ,1.40) |
| 8 | PrEP Navigation App (Implementation) [Sullivan, 2022, (10)] | 2.19 (1.00, 4.32) | PrEP uptake | 40% agreed to participate/receive intervention (1226/3049 participants in this study) | Not applicable – provided independently of care provider. | 84% of people indicated for PrEP in the US have insurance coverage(61)) * 91% of adults have a smartphone(70) (financial barrier) | 1.37 (1.00 ,2.02) |
| 9 | Personalized PrEP Support (Implementation) [Desrosiers, 2019, (11)] | 1.32 (1.10, 1.82) | PrEP uptake | 44% will accept this intervention when offered (11/25 study participants spoke with provider about PrEP within 3-month follow-up) | 93% of US health centers have dedicated staff for HIV outreach and PrEP services(62) | 84% of people indicated for PrEP in the US have insurance coverage(61) | 1.11 (1.03 ,1.28) |
| 10 | PrEP case management (Implementation) [Doblecki-Lewis, 2019, (12)] | 1.38 (1.24, 1.59) | PrEP uptake | 56% agreed to participate/receive intervention (61/110 study participants met eligibility criteria for the study) | 93% of US health centers have dedicated staff for HIV outreach and PrEP services(62) | 84% of people indicated for PrEP in the US have insurance coverage(61) | 1.16 (1.10 ,1.26) |
| 11 | Motivational interviewing for PrEP (Implementation) [Chan, 2021, (13)] | 2.09 (1.32, 2.78) | PrEP uptake | 51% agreed to participate/receive intervention (147/287 people recruited and participated of those approached for this study) | 93% of US health centers have dedicated staff for HIV outreach and PrEP services(62) | 84% of people indicated for PrEP in the US have insurance coverage(61) | 1.43 (1.13 ,1.71) |
| 12 | Interactive Digital PrEP Adherence (Implementation) [Liu, 2019, (14)] | 1.28 (1.03, 1.48) | PrEP adherence | 90% (121/134 of study participants screened were enrolled) | Not applicable – provided independently of care provider. | 91% of adults have a smartphone(70) (financial barrier). Other financial barriers not applied because population is already on PrEP | 1.23 (1.02 ,1.39) |
| 13 | Bidirectional support messages (Implementation) [Fuchs, 2018, (15)] | 1.40 (1.00, 2.80) | PrEP adherence | 68% (38/56 of study participants opted for SMS delivery) | Not applicable – provided independently of care provider. | 91% of adults have a smartphone(70) (financial barrier). Other financial barriers not applied because population is already on PrEP | 1.24 (1.00 ,2.13) |
| 14 | Personalized SMS (Implementation) [Moore, 2018, (16)] | 1.37 (1.00, 1.79) | PrEP adherence | 92% (398/435 of study participants were randomized from screened individuals) | Not applicable – provided independently of care provider. | 91% of adults have a smartphone(70) (financial barrier). Other financial barriers not applied because population is already on PrEP | 1.31 (1.00 ,1.66) |
| 15 | Nurse-led counseling (Implementation) [Mayer, 2017, (17)] | 1.33 (1.16, 1.50) | PrEP adherence | 86% (50/58of study participants randomized from eligible individuals) | 93% of US health centers have dedicated staff for HIV outreach and PrEP services(62) | Financial barrier not applied because population is already on PrEP | 1.26 (1.13 ,1.40) |
| ***Diagnosis & Response - HIV testing interventions*** | | | | | | | |
| 16 | Medicaid Expansion for testing (Structural) [Farkhad, 2021, (18)] | 1.16 (1.08, 1.25) | HIV testing | N/A | N/A | N/A | 1.16 (1.08 ,1.25) |
| 17 | Mobile clinic testing (Structural) [Ellen, 2003, (19)] | 1.12 (1.09, 1.15) | HIV testing | 54.4% (381/700 of study clients sought out HIV testing offered by mobile clinic) | 45% of mobile health units receive federal funding(60) | 100% federally-funded services offered at no cost and low-barrier | 1.03 (1.02 ,1.04) |
| 18 | Federally-funded pharmacy testing (Structural) [Collins, 2018, (20)] | 2.00 (1.40, 2.95) | HIV positivity rate | 90.1% of people are willing to accept free HIV test(71) | 71% (in this study, 1087/1540 tests were conducted in a pharmacy or retail clinic) | 100% federally-funded services offered at no cost and low-barrier | 1.64 (1.26 ,2.25) |
| 19 | PWID mobile van (Structural) [Page KR, 2024,(21)] | 1.20 (0.90,1.73) | HIV testing | 75% (268/360) completed 7-mo follow-up | 45% of mobile health units receive federal funding(60) | 100% federally-funded services offered at no cost and low-barrier | 1.05(0.98, 1.22) |
| 20 | Referral-based testing (Biomedical) [Katz, 2016, (22)] | 1.44 (1.42, 1.47) | HIV testing | 69% of this intervention’s participants accepted the referral from partner services to test for HIV at a clinic (Average between 2008 (62%) of 3253 preintervention participants and 3712 (76%) of 4880 during the intervention received partner services). | No additional adoption barriers, as the intervention is integrated into existing partner care services | 80% (71.6%-87.9% MSM insured in the US, non-Medicaid expansion vs. Medicaid expansion states)(3). | 1.24 (1.23 ,1.25) |
| 21 | Hospital-based testing (Biomedical) (Felsen, 2017, (23)] | 2.38 (2.27, 2.50) | HIV testing | 20% (11%-29% of people had an acute care visit in the last 12 months)(72, 73). | 99% (97.2%-100% of US emergency departments reporting use of an electronic health record system)(72, 74) | No additional insurance barriers, as intervention is offered to those already receiving care in hospital | 1.27 (1.14 ,1.43) |
| 22 | ED-based testing (Biomedical) [Montoy, 2016, (24)] | 1.39 (1.32, 1.45) | HIV testing | 20% (11%-29% of people had an acute care visit in the last 12 months)(72, 73). | 22% (18.7%-26.0% of individuals that were offered hospital-based routine HIV testing in the US(75)) | No additional insurance barriers, as intervention is offered to those already receiving care in hospital | 1.02 (1.01 ,1.02) |
| 23 | Jail/prison-based testing (Biomedical) [Iroh, 2015, (25)] | 1.02 (1.0169, 1.0172) | HIV positivity rate | 81% (acceptance rate of rapid HIV tests in a County jail setting 17035/20947 participants accepted from offer at intake)(76) * 0.4% (1,230,100/333,287,557 percentage of US population in prison or jail(77) from total US population in 2022(78)) | 69% (proportion of detainees offered HIV testing at intake in a County jail)(76) | No additional barriers, as intervention is offered to those already in jail/prison | 1.00 (1.00 ,1.00) |
| 24 | Pharmacy/Retail-based testing (Biomedical) [Weidle, 2014, (26)] | 1.02 (1.01,1.02) | HIV positivity rate | 90.1% of people are willing to accept free HIV test(71) | 71% (1087/1540 tests were conducted in a pharmacy or retail clinic in this study) | 80% (71.6%-87.9% MSM insured in the US, non-Medicaid expansion vs. Medicaid expansion states)(3) | 1.01 (1.00 ,1.01) |
| 25 | CBO testing (Biomedical) [Sanchez, 2014, (27)] | 1.009 (1.009, 1.010) | HIV positivity rate | 43% (5878/13827 of study patients who were offered screening accepted it) | 13% (101,239/799,573 of tests in this study were conducted in community) | 100% federally-funded services offered at no cost and low-barrier | 1.00 (1.00 ,1.00) |
| 26 | Internet self-testing kit (Biomedical) [MacGowan, 2020, (28)] | 2.38 (1.90, 2.98) | HIV testing | 90.1% of people are willing to accept free HIV test(71) | Not applicable – provided independently of care provider. | 80% (71.6%-87.9% MSM insured in the US, non-Medicaid expansion vs. Medicaid expansion states)(3) | 1.99 (1.58 ,2.57) |
| 27 | Multi-platform self-testing kit distribution (Biomedical) [Eshun-Wilson, 2021, (29)] | 1.55 (1.01, 2.76) | HIV testing | 90.1% of people are willing to accept free HIV test(71) | Not applicable – provided independently of care provider. | 100% Services offered at no cost and low barrier, provided by community | 1.50 (1.01 ,2.59) |
| 28 | Partner care (Biomedical) [Dalal, 2017, (30)] | 1.46 (1.22, 1.75) | HIV testing | 50% (people with new HIV diagnoses in the US were interviewed for partner services***)(79) | 100% Assume no additional adoption barriers as the intervention is added into existing partner care services | 80% (71.6%-87.9% MSM insured in the US, non-Medicaid expansion vs. Medicaid expansion states)(3) | 1.18 (1.08 ,1.33) |
| 29 | Phylogenetic outbreak response (Implementation) [McClung, 2021, (31)] | 1.03 (1.01, 1.06) | HIV positivity rate | 75% (response rate to partner services call(80) | 93% of US health centers have dedicated staff for HIV outreach and PrEP services(62) | 80% (71.6%-87.9% MSM insured in the US, non-Medicaid expansion vs. Medicaid expansion states)(3) | 1.02 (1.01 ,1.04) |
| 30 | Peer-Led HIV Testing (Implementation) [Young, 2013, (32)] | 2.00 (1.11, 7.88) | HIV testing | 92% (112/122 participants randomized to the intervention from those screened) | Not applicable – provided independently of care provider. | 91% of adults have a smartphone(70) (financial barrier). No insurance barriers, as free testing is part of intervention | 1.84 (1.09 ,6.75) |
| 31 | Test Awareness Campaign (Implementation) [Habarta, 2017, (33)] | 1.38 (1.11, 1.73) | HIV testing | 43% (303/702 reported intervention exposure) | Not applicable – provided independently of care provider. | 80% (71.6%-87.9% MSM insured in the US, non-Medicaid expansion vs. Medicaid expansion states)(3) | 1.13 (1.03 ,1.28) |
| 32 | Social media health educator (Implementation) [Rhodes, 2016, (34)] | 1.52 (1.44, 1.60) | HIV testing | 27% (343/1292 were willing to participate in the intervention) | Not applicable – provided independently of care provider. | 80% (71.6%-87.9% MSM insured in the US, non-Medicaid expansion vs. Medicaid expansion states)(3) | 1.11 (1.08 ,1.14) |
| 33 | Routine Testing Training (Implementation) [Lubelchek, 2013, (35)] | 2.06 (1.40, 2.86) | HIV testing | N/A | 83% providers completed the survey * 26% providers attended the training in this intervention | 80% (71.6%-87.9% MSM insured in the US, non-Medicaid expansion vs. Medicaid expansion states)(3) | 1.18 (1.06 ,1.35) |
| 34 | Personalized testing options (Implementation) [Frye, 2020, (36)] | 1.85 (1.80, 2.02) | HIV testing | 63.8% of MSM reported want to use  sexual health information via app(81) * 66.5% of MSM reported using dating apps(82) | Not applicable – provided independently of care provider. | 80% (71.6%-87.9% MSM insured in the US, non-Medicaid expansion vs. Medicaid expansion states)(3) | 1.29 (1.24 ,1.38) |
| ***Treatment & Response – ART interventions*** | | | | | | | |
| 35 | Medicaid Expansion for ART (Structural) [Furl, 2018, (37)] | 1.45 (1.34, 1.58) | Viral suppression | N/A | N/A | N/A | 1.45 (1.34 ,1.58) |
| 36 | Extend ADAP Recertification (Structural) [(Erly, 2023, (38)] | 1.14 (1.10, 1.18) | Viral suppression | 50% of PLHIV are enrolled in Ryan White(83) | N/A | No additional insurance barriers, as intervention is offered to those enrolled in Ryan White | 1.07 (1.05 ,1.09) |
| 37 | Tele HIV Care & Support (Structural) [Salgado, 2021, (39)] | 1.04 (1.03, 1.05) | Viral suppression | 15% (1041/6977 Georgia Ryan White HIV/AIDS Program clients used telehealth services during intervention)  * 50% of PLHIV are enrolled in Ryan White(83) | 86.9% of U.S. hospitals offered telehealth services(84) | No additional insurance barriers, as intervention is offered to those enrolled in Ryan White | 1.00 (1.00 ,1.00) |
| 38 | Specialty telehealth in primary care (Structural) [Ohl, 2019, (40)] | 1.14 (1.01, 1.30) | Viral suppression | 13% (120/925 individuals in this study used telehealth when it was available) * 3% of PLHIV received Veteran Affairs service(85) | 86.9% of U.S. hospitals offered telehealth services(84) | No additional insurance barriers, as intervention is offered to those receiving Veteran Affairs services | 1.00 (1.00 ,1.00) |
| 39 | Standardized HIV Care Protocol (Structural) [Halperin, 2018, (41)] | 1.82 (1.49, 2.00) | ART initiation | 92% (71/77 newly diagnosed were linked to care and started ART within 72h in this study) (41) (41) (41) (41) (41) (40) (40) (40) (40) (40) (40) (40) (40) (46) (46) (54) (54) (54) (54) (54) | 93% of US health centers have dedicated staff for HIV outreach and PrEP services(62) | 81% of US PLHIV have adequate geographic access to HIV care (within 30 min drive time)(86) * 80% (71.6%-87.9% MSM insured in the US, non-Medicaid expansion vs. Medicaid expansion states)(3) | 1.45 (1.24 ,1.61) |
| 40 | Extended ART prescriptions (Structural) [McGinnis, 2021, (42)] | 1.053 (1.052, 1.053) | ART adherence | 3% of PLHIV received Veteran Affairs service(85) | 100% No adoption barriers, as regulatory change is required | No additional insurance barriers, as intervention is offered to those receiving Veteran Affairs services | 1.02 (1.02 ,1.02) |
| 41 | ART mobile van (Structural) [Altice, 2007, (43)] | 1.39 (1.08, 1.59) | Viral suppression | 84% (74/88 patients accepted intervention) | 45% of mobile health units receive federal funding(60) | 80% (71.6%-87.9% MSM insured in the US, non-Medicaid expansion vs. Medicaid expansion states)(3) | 1.12 (1.02 ,1.20) |
| 42 | Community-pharmacist collaboration (Structural) [Byrd, 2020, (44)] | 1.23 (1.21, 1.25) | Viral suppression | 93% (714/765 filled prescriptions at project pharmacy in this study) | 22% (11/50 states implementing HIV specialty pharmacies)(87) | 80% (71.6%-87.9% MSM insured in the US, non-Medicaid expansion vs. Medicaid expansion states)(3) | 1.04 (1.03 ,1.05) |
| 43 | ART case management (Structural) [Gardner, 2005, (45)] | 1.41 (1.10, 1.60) | ART initiation | 82% of PLHIV were linked to care within a month(88) | 93% of US health centers have dedicated staff for HIV outreach and PrEP services(62) | 81% of US PLHIV have adequate geographic access to HIV care (within 30 min drive time)(86) * 80% (71.6%-87.9% MSM insured in the US, non-Medicaid expansion vs. Medicaid expansion states)(3) | 1.20 (1.04 ,1.33) |
| 44 | ART Re-linkage program (Structural) [Bove, 2015, (46)] | 1.70 (1.20, 2.30) | ART re-initiation | 24% (38/157 patients in this study were successfully contacted of those eligible for re-linkage) | 93% of US health centers have dedicated staff for HIV outreach and PrEP services(62) | 81% of US PLHIV have adequate geographic access to HIV care (within 30 min drive time)(86) * 80% (71.6%-87.9% MSM insured in the US, non-Medicaid expansion vs. Medicaid expansion states)(3) | 1.10 (1.03 ,1.21) |
| 45 | Data to Care re-linkage (Structural) [Fanfair, 2021, (47)] | 1.23 (1.21, 1.25) | ART re-initiation | 10.63% (1893 was randomized among 17809 assessed for eligibility) | 93% of US health centers have dedicated staff for HIV outreach and PrEP services(62) | 81% of US PLHIV have adequate geographic access to HIV care (within 30 min drive time)(86) * 80% (71.6%-87.9% MSM insured in the US, non-Medicaid expansion vs. Medicaid expansion states)(3) | 1.02(1.01, 1.04) |
| 46 | ART Outbreak response platform (Structural) [End Stigma, End HIV Alliance, 2020, (48)] | 1.63 (1.40, 1.75) | ART initiation | 86% (30/35 PLHIV in this study were linked or relinked to care) | 93% of US health centers have dedicated staff for HIV outreach and PrEP services(62) | 81% of US PLHIV have adequate geographic access to HIV care (within 30 min drive time)(86) * 80% (71.6%-87.9% MSM insured in the US, non-Medicaid expansion vs. Medicaid expansion states)(3) | 1.33 (1.19 ,1.43) |
| 47 | ART care coordination (Biomedical) [Robertson, 2018, (49)] | 1.10 (1.07, 1.13) | Viral suppression | 92% (71/77 newly diagnosed were linked to care and started ART within 72h)(41) | 26% (20.0%-33.0% of clinics receiving RWHAP funding and offering case management)(72, 89) | 56% (37.7%-75.0% of US PLHIV were eligible for Ryan White HIV/AIDS Program (RWHAP) Part A services)(72, 90-96) | 1.01 (1.00 ,1.03) |
| 48 | Long-acting ART (Biomedical) [Rana, 2024, (50)] | 1.22 (1.06, 1.45) | Viral suppression | N/A | 100% Assume most providers who already prescribed ART can prescribe long-acting injectable ART | 5% of RWHAP Part C clinics developed procedures and policies to implement long-acting ART prescribing(97) | 1.01 (1.00 ,1.02) |
| 49 | RAPID ART (Biomedical) [Pilcher, 2017, (51)] | 1.47 (1.30, 2.17) | ART initiation | 82% of PLHIV were linked to care within a month(88) | 93% of US health centers have dedicated staff for HIV outreach and PrEP services(62) | 81% of US PLHIV have adequate geographic access to HIV care (within 30 min drive time)(86) * 80% (71.6%-87.9% MSM insured in the US, non-Medicaid expansion vs. Medicaid expansion states)(3) | 1.23 (1.13 ,1.64) |
| 50 | HIV Care & Support App (Implementation) [Zurlo, 2020, (52)] | 1.90 (1.66, 2.20) | ART adherence | 91% (84/92 study participants accepted intervention at 6 months) | Not applicable – provided independently of care provider. | 91% of adults have a smartphone(70) (financial barrier) * 80% (71.6%-87.9% MSM insured in the US, non-Medicaid expansion vs. Medicaid expansion states)(3) | 1.60 (1.39 ,1.88) |
| 51 | Clinic-based Video Counseling (Implementation) [Lewis, 2022, (53)] | 1.14 (1.00, 1.29) | Viral suppression | 56% (799/1419 study participants met inclusion criteria, accepted and were randomized from those eligible) | 93% of US health centers have dedicated staff for HIV outreach and PrEP services(62) | 81% of US PLHIV have adequate geographic access to HIV care (within 30 min drive time)(86) * 80% (71.6%-87.9% MSM insured in the US, non-Medicaid expansion vs. Medicaid expansion states)(3) | 1.05 (1.00 ,1.11) |
| 52 | Clinic-based education app (Implementation) [Dillingham, 2018, (54)] | 1.85 (1.60, 2.19) | Viral suppression | 89% (77/87 study participants enrolled from those eligible) | 93% of US health centers have dedicated staff for HIV outreach and PrEP services(62) | 81% of US PLHIV have adequate geographic access to HIV care (within 30 min drive time)(86) * 80% (71.6%-87.9% MSM insured in the US, non-Medicaid expansion vs. Medicaid expansion states)(3) | 1.45 (1.29 ,1.70) |
| 53 | Social media health educator for ART (Implementation) [Tanner, 2018, (55)] | 1.45 (1.32, 1.60) | Viral suppression | 81% (91/113 study participants enrolled from those invited to participate) | Not applicable – provided independently of care provider. | 80% (71.6%-87.9% MSM insured in the US, non-Medicaid expansion vs. Medicaid expansion states)(3) | 1.29 (1.18 ,1.42) |
| 54 | EMR alert (Implementation) [Robbins, 2012, (56)] | 1.45 (1.11, 1.89) | ART adherence | 76% of people with HIV received HIV medical care within past 12 months in 2023(98) | 78% of office-based physicians use EMRs(99) | 80% (71.6%-87.9% MSM insured in the US, non-Medicaid expansion vs. Medicaid expansion states)(3) | 1.21 (1.05 ,1.46) |
| 55 | Incentivized walk-in clinic (Implementation) [Dombrowski, 2019, (57) | 3.2 (1.8, 5.9) | Viral suppression | 5% of adults diagnosed with HIV are virally unsuppressed and poorly engaged in care (8% who did not receive enough care * 61.2% who are unsuppressed)(100) | 93% of US health centers have dedicated staff for HIV outreach and PrEP services(62) | 81% of US PLHIV have adequate geographic access to HIV care (within 30 min drive time)(86) * 80% (71.6%-87.9% MSM insured in the US, non-Medicaid expansion vs. Medicaid expansion states)(3) | 1.07 (1.02 ,1.16) |
| 56 | E-VOLUTION mobile health (mHealth) intervention with a human support element. (Implementation) [Gerke, D. R., 2023, (58)] | 1.43 (1.12, 1.84) | Viral suppression | 62% of participants engaged in monthly text-message exchanges with medical case managers | Not applicable – provided independently of care provider. | 91% of adults have a smartphone(70) (financial barrier). Other financial barriers not applied because population is already on ART. | 1.19 (1.05, 1.42) |
| 57 | RISE peer counselor (structural) [Bogart 2022,(59)] | 1.28 (1.04,1.46) | ART adherence | 91.8% received at least 1 intervention session | 93% of US health centers have dedicated staff for HIV outreach and PrEP services(62) | 81% of US PLHIV have adequate geographic access to HIV care (within 30 min drive time)(86) * 80% (71.6%-87.9% MSM insured in the US, non-Medicaid expansion vs. Medicaid expansion states)(3) | 1.16 (1.02, 1.28) |
| Abbreviations: PrEP: pre-exposure prophylaxis; MAT: medication assisted treatment (for opioid use disorder e.g. methadone, buprenorphine); ART: antiretroviral treatment; MSM: men who have sex with men; PWID: people who inject drugs; EBI: evidence-based intervention; EI: evidence-informed intervention; STI: sexually transmitted infection; ADAP: AIDS Drug Assistance Program; SSP: syringe service programs; PLHIV: people living with HIV; YBMSM: young black men who have sex with men; RAPID: Rapid ART Program for Individuals with an HIV Diagnosis; RWHAP: Ryan White HIV/AIDS Program; aPR: adjusted prevalence ratio; OR: odds ratio; RR: risk ratio. TFV-DP: Tenofovir-Diphosphate; EMR: electronic medical record; MSM: men who have sex with men; aOR: adjusted odds ratio; aRR: adjusted risk ratio; CDC: Centers for Disease Control and Prevention; aRRR: adjusted relative risk ratio. | | | | | | | |
| *** of CDC-funded tests includes missing/invalid data in denominator. | | | | | | | |

**References**

1. Grant RL. Converting an odds ratio to a range of plausible relative risks for better communication of research findings. Bmj. 2014;348:f7450.

2. Holmberg MJ, Andersen LW. Estimating Risk Ratios and Risk Differences: Alternatives to Odds Ratios. JAMA. 2020;324(11):1098-9.

3. Baugher AR, Finlayson T, Lewis R, Sionean C, Whiteman A, Wejnert C. Health Care Coverage and Preexposure Prophylaxis (PrEP) Use Among Men Who Have Sex With Men Living in 22 US Cities With vs Without Medicaid Expansion, 2017. Am J Public Health. 2021;111(4):743-51.

4. Doblecki-Lewis S, Johnson A, Klose K, King K, Narcisse G, Butts S, et al. An observational cohort study evaluating PrEP reach, engagement and persistence through a community-based mobile clinic in Miami-Dade County, Florida. J Int AIDS Soc. 2024;27(10):e26362.

5. Coleman M, Hodges A, Henn S, Lambert CC. Integrated Pharmacy and PrEP Navigation Services to Support PrEP Uptake: A Quality Improvement Project. J Assoc Nurses AIDS Care. 2020;31(6):685-92.

6. Aspinall EJ, Nambiar D, Goldberg DJ, Hickman M, Weir A, Van Velzen E, et al. Are needle and syringe programmes associated with a reduction in HIV transmission among people who inject drugs: a systematic review and meta-analysis. Int J Epidemiol. 2014;18(11):2144-55.

7. MacArthur GJ, Minozzi S, Martin N. Opiate substitution treatment and HIV transmission in people who inject drugs: systematic review and meta-analysis. BMJ. 2012(345):e5945.

8. Stansfield SE, Moore M, Boily MC, Hughes JP, Donnell DJ, Dimitrov DT. Estimating benefits of using on-demand oral prep by MSM: A comparative modeling study of the US and Thailand. EClinicalMedicine. 2023;56:101776.

9. Landovitz RJ, Donnell D, Clement ME, Hanscom B, Cottle L, Coelho L, et al. Cabotegravir for HIV Prevention in Cisgender Men and Transgender Women. N Engl J Med. 2021;385(7):595-608.

10. Sullivan PS, Stephenson R, Hirshfield S, Mehta CC, Zahn R, Bauermeister JA, et al. Behavioral Efficacy of a Sexual Health Mobile App for Men Who Have Sex With Men: Randomized Controlled Trial of Mobile Messaging for Men. J Med Internet Res. 2022;24(2):e34574.

11. Desrosiers A, Levy M, Dright A, Zumer M, Jallah N, Kuo I, et al. A Randomized Controlled Pilot Study of a Culturally-Tailored Counseling Intervention to Increase Uptake of HIV Pre-exposure Prophylaxis Among Young Black Men Who Have Sex with Men in Washington, DC. AIDS Behav. 2019;23(1):105-15.

12. Doblecki-Lewis S, Butts S, Botero V, Klose K, Cardenas G, Feaster D. A Randomized Study of Passive versus Active PrEP Patient Navigation for a Heterogeneous Population at Risk for HIV in South Florida. J Int Assoc Provid AIDS Care. 2019;18:2325958219848848.

13. Chan PA, Nunn A, van den Berg JJ, Cormier K, Sowemimo-Coker G, Napoleon SC, et al. A Randomized Trial of a Brief Behavioral Intervention for PrEP Uptake Among Men Who Have Sex With Men at Increased Risk for HIV Infection. J Acquir Immune Defic Syndr. 2021;87(3):937-43.

14. Liu AY, Vittinghoff E, von Felten P, Rivet Amico K, Anderson PL, Lester R, et al. Randomized Controlled Trial of a Mobile Health Intervention to Promote Retention and Adherence to Preexposure Prophylaxis Among Young People at Risk for Human Immunodeficiency Virus: The EPIC Study. Clin Infect Dis. 2019;68(12):2010-7.

15. Fuchs JD, Stojanovski K, Vittinghoff E, McMahan VM, Hosek SG, Amico KR, et al. A Mobile Health Strategy to Support Adherence to Antiretroviral Preexposure Prophylaxis. AIDS Patient Care STDS. 2018;32(3):104-11.

16. Moore DJ, Jain S, Dubé MP, Daar ES, Sun X, Young J, et al. Randomized Controlled Trial of Daily Text Messages to Support Adherence to Preexposure Prophylaxis in Individuals at Risk for Human Immunodeficiency Virus: The TAPIR Study. Clin Infect Dis. 2018;66(10):1566-72.

17. Mayer KH, Safren SA, Elsesser SA, Psaros C, Tinsley JP, Marzinke M, et al. Optimizing Pre-Exposure Antiretroviral Prophylaxis Adherence in Men Who Have Sex with Men: Results of a Pilot Randomized Controlled Trial of "Life-Steps for PrEP". AIDS Behav. 2017;21(5):1350-60.

18. Fayaz Farkhad B, Holtgrave DR, Albarracín D. Effect of Medicaid Expansions on HIV Diagnoses and Pre-Exposure Prophylaxis Use. Am J Prev Med. 2021;60(3):335-42.

19. Ellen JM, Bonu S, Arruda JS, Ward MA, Vogel R. Comparison of clients of a mobile health van and a traditional STD clinic. J Acquir Immune Defic Syndr. 2003;32(4):388-93.

20. Collins B, Bronson H, Elamin F, Yerkes L, Martin E. The "No Wrong Door" Approach to HIV Testing: Results From a Statewide Retail Pharmacy-Based HIV Testing Program in Virginia, 2014-2016. Public Health Rep. 2018;133(2_suppl):34s-42s.

21. Page KR, Weir BW, Zook K, Rosecrans A, Harris R, Grieb SM, et al. Integrated care van delivery of evidence-based services for people who inject drugs: A cluster-randomized trial. Addiction. 2024;119(7):1276-88.

22. Katz DA, Dombrowski JC, Kerani RP, Aubin MR, Kern DA, Heal DD, et al. Integrating HIV Testing as an Outcome of STD Partner Services for Men Who Have Sex with Men. AIDS Patient Care STDS. 2016;30(5):208-14.

23. Felsen UR, Cunningham CO, Heo M, Futterman DC, Weiss JM, Zingman BS. Expanded HIV Testing Strategy Leveraging the Electronic Medical Record Uncovers Undiagnosed Infection Among Hospitalized Patients. J Acquir Immune Defic Syndr. 2017;75(1):27-34.

24. Montoy JC, Dow WH, Kaplan BC. Patient choice in opt-in, active choice, and opt-out HIV screening: randomized clinical trial. Bmj. 2016;532:h6895.

25. Iroh PA, Mayo H, Nijhawan AE. The HIV Care Cascade Before, During, and After Incarceration: A Systematic Review and Data Synthesis. Am J Public Health. 2015;105(7):e5-16.

26. Weidle PJ, Lecher S, Botts LW, Jones L, Spach DH, Alvarez J, et al. HIV testing in community pharmacies and retail clinics: a model to expand access to screening for HIV infection. J Am Pharm Assoc (2003). 2014;54(5):486-92.

27. Sanchez TH, Sullivan PS, Rothman RE, Brown EH, Fitzpatrick LK, Wood AF, et al. A Novel Approach to Realizing Routine HIV Screening and Enhancing Linkage to Care in the United States: Protocol of the FOCUS Program and Early Results. JMIR Res Protoc. 2014;3(3):e39.

28. MacGowan RJ, Chavez PR, Borkowf CB, Owen SM, Purcell DW, Mermin JH, et al. Effect of Internet-Distributed HIV Self-tests on HIV Diagnosis and Behavioral Outcomes in Men Who Have Sex With Men: A Randomized Clinical Trial. JAMA Intern Med. 2020;180(1):117-25.

29. Eshun-Wilson I, Jamil MS, Witzel TC, Glidded DV, Johnson C, Le Trouneau N, et al. A Systematic Review and Network Meta-analyses to Assess the Effectiveness of Human Immunodeficiency Virus (HIV) Self-testing Distribution Strategies. Clin Infect Dis. 2021;73(4):e1018-e28.

30. Dalal S, Johnson C, Fonner V, Kennedy CE, Siegfried N, Figueroa C, et al. Improving HIV test uptake and case finding with assisted partner notification services. Aids. 2017;31(13):1867-76.

31. McClung RP, Atkins AD, Kilkenny M, Bernstein KT, Willenburg KS, Weimer M, et al. Response to a Large HIV Outbreak, Cabell County, West Virginia, 2018-2019. Am J Prev Med. 2021;61(5 Suppl 1):S143-s50.

32. Young SD, Cumberland WG, Lee SJ, Jaganath D, Szekeres G, Coates T. Social networking technologies as an emerging tool for HIV prevention: a cluster randomized trial. Ann Intern Med. 2013;159(5):318-24.

33. Habarta N, Boudewyns V, Badal H, Johnston J, Uhrig J, Green D, et al. CDC'S Testing Makes Us Stronger (TMUS) Campaign: Was Campaign Exposure Associated With HIV Testing Behavior Among Black Gay and Bisexual Men? AIDS Educ Prev. 2017;29(3):228-40.

34. Rhodes SD, McCoy TP, Tanner AE, Stowers J, Bachmann LH, Nguyen AL, et al. Using Social Media to Increase HIV Testing Among Gay and Bisexual Men, Other Men Who Have Sex With Men, and Transgender Persons: Outcomes From a Randomized Community Trial. Clin Infect Dis. 2016;62(11):1450-3.

35. Lubelchek RJ, Hotton AL, Taussig D, Amarathithada D, Gonzalez M. Scaling up routine HIV testing at specialty clinics: assessing the effectiveness of an academic detailing approach. J Acquir Immune Defic Syndr. 2013;64 Suppl 1(0 1):S14-9.

36. Frye V, Nandi V, Hirshfield S, Chiasson MA, Wilton L, Usher D, et al. Brief Report: Randomized Controlled Trial of an Intervention to Match Young Black Men and Transwomen Who Have Sex With Men or Transwomen to HIV Testing Options in New York City (All About Me). J Acquir Immune Defic Syndr. 2020;83(1):31-6.

37. Furl R, Watanabe-Galloway S, Lyden E, Swindells S. Determinants of facilitated health insurance enrollment for patients with HIV disease, and impact of insurance enrollment on targeted health outcomes. BMC Infect Dis. 2018;18(1):132.

38. Erly SJ, Khosropour CM, Hajat A, Sharma M, Reuer JR, Dombrowski JC. AIDS Drug Assistance Program disenrollment is associated with loss of viral suppression beyond differences in homelessness, mental health, and substance use disorders: An evaluation in Washington state 2017-2019. PLoS One. 2023;18(5):e0285326.

39. Salgado S, Felzien G, Brumbeloe J. Georgia Leverages Telehealth to Expand HIV Care Management in Underserved Areas. American Journal of Preventive Medicine. 2021;61(5, Supplement 1):S55-S9.

40. Ohl ME, Richardson K, Rodriguez-Barradas MC, Bedimo R, Marconi V, Morano JP, et al. Impact of Availability of Telehealth Programs on Documented HIV Viral Suppression: A Cluster-Randomized Program Evaluation in the Veterans Health Administration. Open Forum Infect Dis. 2019;6(6):ofz206.

41. Halperin J, Butler I, Conner K, Myers L, Holm P, Bartram L, et al. Linkage and Antiretroviral Therapy Within 72 Hours at a Federally Qualified Health Center in New Orleans. AIDS Patient Care STDS. 2018;32(2):39-41.

42. McGinnis KA, Skanderson M, Justice AC, Akgün KM, Tate JP, King JT, Jr., et al. HIV care using differentiated service delivery during the COVID-19 pandemic: a nationwide cohort study in the US Department of Veterans Affairs. J Int AIDS Soc. 2021;24 Suppl 6(Suppl 6):e25810.

43. Altice FL, Maru DS, Bruce RD, Springer SA, Friedland GH. Superiority of directly administered antiretroviral therapy over self-administered therapy among HIV-infected drug users: a prospective, randomized, controlled trial. Clin Infect Dis. 2007;45(6):770-8.

44. Byrd KK, Hou JG, Bush T, Hazen R, Kirkham H, Delpino A, et al. Adherence and Viral Suppression Among Participants of the Patient-centered Human Immunodeficiency Virus (HIV) Care Model Project: A Collaboration Between Community-based Pharmacists and HIV Clinical Providers. Clin Infect Dis. 2020;70(5):789-97.

45. Gardner LI, Metsch LR, Anderson-Mahoney P, Loughlin AM, del Rio C, Strathdee S, et al. Efficacy of a brief case management intervention to link recently diagnosed HIV-infected persons to care. AIDS. 2005;19(4):423-31.

46. Bove JM, Golden MR, Dhanireddy S, Harrington RD, Dombrowski JC. Outcomes of a Clinic-Based Surveillance-Informed Intervention to Relink Patients to HIV Care. J Acquir Immune Defic Syndr. 2015;70(3):262-8.

47. Fanfair RN, Khalil G, Williams T, Brady K, DeMaria A, Villanueva M, et al. The Cooperative Re-Engagement Controlled trial (CoRECT): A randomised trial to assess a collaborative data to care model to improve HIV care continuum outcomes. Lancet Reg Health Am. 2021;3:100057.

48. US Centers for Disease Control and Prevention. CDR Community Spotlights: San Antonio, Texas. 2021. [Available from: <https://www.cdc.gov/hiv/policies/cdr/spotlights/texas.html> (Accessed September 12, 2024).

49. Robertson MM, Waldron L, Robbins RS, Chamberlin S, Penrose K, Levin B, et al. Using Registry Data to Construct a Comparison Group for Programmatic Effectiveness Evaluation: The New York City HIV Care Coordination Program. Am J Epidemiol. 2018;187(9):1980-9.

50. Rana AI, Bao Y, Zheng L, Sieczkarski S, Lake JE, Fichtenbaum CJ, et al., editors. Long-Acting Injectable CAB/RPV is Superior to Oral ART in PWH With Adherence Challenges: ACTG A5359. Conference presentation. Abstract 212. Oral session 14. Conference on Retroviruses and Opportunistic Infections; 2024; Denver, CO.

51. Pilcher CD, Ospina-Norvell C, Dasgupta A, Jones D, Hartogensis W, Torres S, et al. The Effect of Same-Day Observed Initiation of Antiretroviral Therapy on HIV Viral Load and Treatment Outcomes in a US Public Health Setting. J Acquir Immune Defic Syndr. 2017;74(1):44-51.

52. Zurlo J, Du P, Haynos A, Collins V, Eshak T, Whitener C. OPT-In For Life: A Mobile Technology-Based Intervention to Improve HIV Care Continuum for Young Adults Living With HIV. Health Promot Pract. 2020;21(5):727-37.

53. Lewis MA, Harshbarger C, Bann C, Marconi VC, Somboonwit C, Piazza MD, et al. Effectiveness of an Interactive, Highly Tailored "Video Doctor" Intervention to Suppress Viral Load and Retain Patients With HIV in Clinical Care: A Randomized Clinical Trial. J Acquir Immune Defic Syndr. 2022;91(1):58-67.

54. Dillingham R, Ingersoll K, Flickinger TE, Waldman AL, Grabowski M, Laurence C, et al. PositiveLinks: A Mobile Health Intervention for Retention in HIV Care and Clinical Outcomes with 12-Month Follow-Up. AIDS Patient Care STDS. 2018;32(6):241-50.

55. Tanner AE, Song EY, Mann-Jackson L, Alonzo J, Schafer K, Ware S, et al. Preliminary Impact of the weCare Social Media Intervention to Support Health for Young Men Who Have Sex with Men and Transgender Women with HIV. AIDS Patient Care STDS. 2018;32(11):450-8.

56. Robbins GK, Lester W, Johnson KL, Chang Y, Estey G, Surrao D, et al. Efficacy of a clinical decision-support system in an HIV practice: a randomized trial. Ann Intern Med. 2012;157(11):757-66.

57. Dombrowski JC, Galagan SR, Ramchandani M, Dhanireddy S, Harrington RD, Moore A, et al. HIV Care for Patients With Complex Needs: A Controlled Evaluation of a Walk-In, Incentivized Care Model. Open Forum Infectious Diseases. 2019;6(7):ofz294.

58. Gerke DR, Glotfelty J, Slovacek S, Freshman M, Schlueter J, Plax K. More than just Reminders: Using text Messaging to Improve HIV care Outcomes Among Youth and Young Adults Living with HIV. AIDS Behav. 2023;27(9):2988-96.

59. Bogart LM, Mutchler MG, Goggin K, Ghosh-Dastidar M, Klein DJ, Saya U, et al. Randomized Controlled Trial of Rise, A Community-Based Culturally Congruent Counseling Intervention to Support Antiretroviral Therapy Adherence Among Black/African American Adults Living with HIV. AIDS Behav. 2023;27(5):1573-86.

60. Malone NC, Williams MM, Smith Fawzi MC, Bennet J, Hill C, Katz JN, et al. Mobile health clinics in the United States. International Journal for Equity in Health. 2020;19(1):40.

61. Bonacci RA, Van Handel M, Huggins R, Inusah S, Smith DK. Estimated Uncovered Costs For HIV Preexposure Prophylaxis In The US, 2018. Health Aff (Millwood). 2023;42(4):546-55.

62. US Department of Health and Human Services. Health Centers Provide HIV Services to More than 600,000 People in EHE Jurisdictions in Just 8 Months 2021 [Available from: <https://www.hiv.gov/blog/health-centers-provide-hiv-services-more-600000-people-ehe-jurisdictions-just-8-months>.

63. McAteer J, Welch A, Tuazon E, Paone D. Syringe service programs in New York City. Epi Data Brief [Internet]. June 2019 June 17, 2025; 110. Available from: <https://www.nyc.gov/assets/doh/downloads/pdf/epi/databrief110.pdf>.

64. Ray BR, Humphrey JL, Patel SV, Akiba CF, Bluthenthal RN, Tookes H, et al. Comparing harm reduction and overdose response services between community-based and public health department syringe service programmes using a national cross-sectional survey. Lancet Reg Health Am. 2024;34:100757.

65. Legislative Analysis and Public Policy Association. Syringe Services Programs: Summary of State Laws. June 2022. [Available from: <https://legislativeanalysis.org/wp-content/uploads/2022/06/Syringe-Services-Programs-Summary-of-State-Laws.pdf>. Accessed September 9, 2024.

66. Winstanley EL, Thacker EP, Choo LY, Lander LR, Berry JH, Tofighi B. Patient-reported problems filling buprenorphine prescriptions and motivations for illicit use. Drug and Alcohol Dependence Reports. 2022;5:100091.

67. Hartung DM, Voss RW, Bailey SR, Huguet N, Muench J. Changes in Buprenorphine Prescribing in Community Health Centers. 2024(2689-0186 (Electronic)).

68. Martin K. Is Treatment for Opioid Use Disorder Affordable for Those with Public or Private Health Coverage? : Foundation for Opioid Response Efforts; 2021 [Available from: <https://forefdn.org/wp-content/uploads/2021/11/fore-moud-insured.pdf>.

69. US Centers for Disease Control and Prevention. Clinical Guidance for PrEP February 10, 2025 [Available from: <https://www.cdc.gov/hivnexus/hcp/prep/index.html>.

70. Pew Research Center. Mobile Fact Sheet November 13, 2024 [Available from: <https://www.pewresearch.org/internet/fact-sheet/mobile/>.

71. Crawford ND, Harrington KRV, Chandra C, Alohan DI, Quamina A, Beck O, et al. Feasibility of reaching populations at high risk for HIV in community pharmacies. J Am Pharm Assoc (2003). 2024;64(6):102239.

72. Krebs E, Zang X, Enns B, Min JE, Behrends CN, Del Rio C, et al. The impact of localized implementation: determining the cost-effectiveness of HIV prevention and care interventions across six United States cities. Aids. 2020;34(3):447-58.

73. National Center for Health Statistics. National Health Interview Survey, 2015. P*ublic-use data file and documentation Available from URL:*[*http://wwwcdcgov/nchs/nhis/quest_data_related_1997_forwardhtm*](http://wwwcdcgov/nchs/nhis/quest_data_related_1997_forwardhtm) *[accessed June 19, 2018] 2*016.

74. Centers for Disease Control and Prevention (CDC). NAMCS/NHAMCS, 2015—Questionnaires, Datasets, and Related Documentation. Atlanta, GA: U.S. Department of Health and Human Services; 2017.

75. Elgalib A, Fidler S, Sabapathy K. Hospital-based routine HIV testing in high-income countries: a systematic literature review. HIV Medicine. 2018;19(3):195-205.

76. Spaulding AC, Kim MJ, Corpening KT, Carpenter T, Watlington P, Bowden CJ. Establishing an HIV Screening Program Led by Staff Nurses in a County Jail. J Public Health Manag Pract. 2015;21(6):538-45.

77. Carson EA, Kluckow R. Prisoners in 2022 – Statistical Tables2023 June 18, 2025. Available from: <https://bjs.ojp.gov/library/publications/prisoners-2022-statistical-tables>.

78. United States Census Bureau. Growth in U.S. Population Shows Early Indication of Recovery Amid COVID-19 Pandemic: United States Census Bureau; December 22, 2022 [Available from: <https://www.census.gov/newsroom/press-releases/2022/2022-population-estimates.html>.

79. US Centers for Disease Control and Prevention. CDC-Funded HIV Testing in the United States, Puerto Rico, and U.S. Virgin Islands, 2021 Annual HIV Testing Report: US Centers for Disease Control and Prevention; 2024 [Available from: <https://stacks.cdc.gov/view/cdc/149067/cdc_149067_DS1.pdf>.

80. Williams WO, Song W, Huang T, Mulatu MS, Uhl G, Rorie M. HIV Diagnoses Through Partner Services in the United States in 2019 and Opportunities for Improvement. Sex Transm Dis. 2023;50(2):74-8.

81. Sun CJ, Stowers J, Miller C, Bachmann LH, Rhodes SD. Acceptability and feasibility of using established geosocial and sexual networking mobile applications to promote HIV and STD testing among men who have sex with men. AIDS Behav. 2015;19(3):543-52.

82. Hecht J, Zlotorzynska M, Sanchez TH, Wohlfeiler D. Gay Dating App Users Support and Utilize Sexual Health Features on Apps. AIDS Behav. 2022;26(6):2081-90.

83. Health Resources and Services Administration. Ryan White HIV/AIDS Program Achieves Record-Breaking 90.6% Viral Suppression Rate among Its More than 576,000 Clients: U.S. Department of Health and Human Services; December 2, 2024 [Available from: <https://www.hrsa.gov/about/news/press-releases/ryan-white-record-breaking-viral-suppression-rate>.

84. American Hospital Association. Fact Sheet: Telehealth 2025 [Available from: <https://www.aha.org/fact-sheets/2025-02-07-fact-sheet-telehealth>.

85. Dawson L, Kates J, Roberts T. The Health Insurance and Financing Landscape for People with and at Risk for HIV: KFF; May 25, 2023 [Available from: <https://www.kff.org/hivaids/issue-brief/the-health-insurance-and-financing-landscape-for-people-with-and-at-risk-for-hiv/>.

86. Masiano SP, Martin EG, Bono RS, Dahman B, Sabik LM, Belgrave FZ, et al. Suboptimal geographic accessibility to comprehensive HIV care in the US: regional and urban-rural differences. J Int AIDS Soc. 2019;22(5):e25286.

87. US Department of Health & Human Services. Pharmacies Take Action to Address HIV July 31, 2023 [Available from: <https://www.hiv.gov/blog/pharmacies-take-action-to-address-hiv>.

88. US Health and Human Services. America's HIV Epidemic Analysis Dashboard (AHEAD) August 18, 2019 [updated January 8, 2025. Available from: <https://ahead.hiv.gov>.

89. Weiser J, Beer L, Frazier EL, Patel R, Dempsey A, Hauck H, et al. Service Delivery and Patient Outcomes in Ryan White HIV/AIDS Program-Funded and -Nonfunded Health Care Facilities in the United States. JAMA Intern Med. 2015;175(10):1650-9.

90. Health Resources and Services Administration. Ryan White HIV/AIDS Program Annual Client-Level Data Report 2016 2017 [Available from: <http://hab.hrsa.gov/data/data-reports>]. [

91. Center for HIV Surveillance - Epidemiology and Evaluation - Department of Health and Mental Hygiene - Baltimore - MD. Baltimore City Annual HIV Epidemiological Profile 2013 2015 [Available from: <http://health.baltimorecity.gov/sites/default/files/Baltimore_City_HIV_Epidemiological_Profile%202013.pdf>, [Accessed: February 2, 2018].

92. County of Los Angeles Public Health: Division of HIV and STD Programs. 2014 Los Angeles County HIV Cascades and PLWH Estimate 2016 [Available from: <http://publichealth.lacounty.gov/dhsp/Reports/HIV/HIVcascadesHIVEstimate2014.pdf>, [Accessed: February 2, 2018].

93. Florida Department of Health. HIV Surveillance Miami, FL2015 [Available from: <http://miamidade.floridahealth.gov/programs-and-services/infectious-disease-services/hiv-aids-services/hiv-surveillance.html>.

94. Georgia Department of Public Health. Georgia's HIV/AIDS Epidemiology Surveillance Section 2017 [Available from: <https://dph.georgia.gov/georgias-hivaids-epidemiology-surveillance-section>].

95. New York City Department of Health and Mental Hygiene. HIV/AIDS Surveillance and Epidemiology Reports 2017 [Available from: <http://www1.nyc.gov/site/doh/data/data-sets/hiv-aids-surveillance-and-epidemiology-reports.page>].

96. Public Health - Seattle & King County. HIV/STD program 2017 [Available from: <http://www.kingcounty.gov/depts/health/communicable-diseases/hiv-std.aspx>].

97. Tarfa A, Sayles H, Bares SH, Havens JP, Fadul N. Acceptability, Feasibility, and Appropriateness of Implementation of Long-acting Injectable Antiretrovirals: A National Survey of Ryan White Clinics in the United States. Open Forum Infect Dis. 2023;10(7):ofad341.

98. Centers for Disease Control and Prevention. NCHHSTP AtlasPlus [Available from: <https://www.cdc.gov/nchhstp/about/atlasplus.html>.

99. Office of the National Coordinator for Health Information Technology. National Trends in Hospital and Physician Adoption of Electronic Health Records [Health IT Quick-Stat #61:[Available from: <https://www.healthit.gov/data/quickstats/national-trends-hospital-and-physician-adoption-electronic-health-records>.

100. Dasgupta S, Tie Y, Beer L, Fagan J, Weiser J. Barriers to HIV Care by Viral Suppression Status Among US Adults With HIV: Findings From the Centers for Disease Control and Prevention Medical Monitoring Project. J Assoc Nurses AIDS Care. 2021;32(5):561-8.
